# Microglial circDlg1 modulates neuroinflammation by blocking PDE4B ubiquitination-dependent degradation associated with Alzheimer's disease

**DOI:** 10.7150/thno.104709

**Published:** 2025-02-24

**Authors:** Jiyun Shi, Chenghuan Song, Pingao Zhang, Jing Wang, Wanying Huang, Ting Yu, Zijie Wei, Lufeng Wang, Lanxue Zhao, Rui Zhang, Lina Hou, Yongfang Zhang, Hongzhuan Chen, Hao Wang

**Affiliations:** 1 Department of Pharmacology and Chemical Biology, Shanghai Jiao Tong University School of Medicine, Shanghai 200025, China.; 2 Shuguang Lab of Future Health, Shanghai Frontiers Science Center of TCM Chemical Biology, Shuguang Hospital, Shanghai University of Traditional Chinese Medicine, Shanghai 201203, China.; 3 Academy of Integrative Medicine, Shanghai University of Traditional Chinese Medicine, Shanghai 201203, China.; 4 Department of Neurology, Shanghai East Hospital, School of Medicine, Tongji University, Shanghai 200120, China.

**Keywords:** Alzheimer's disease, neuroinflammation, microglia activation, circDlg1, phosphodiesterase 4b

## Abstract

**Background:** Abnormal activation of microglia occurs in the early stage of Alzheimer's disease (AD) and leads to subsequent neuroinflammation and major AD pathologies. Circular RNAs (circRNAs) are emerging as great potential therapeutic targets in AD. However, the extent of circRNAs entwined and the underlying mechanism in microglia-driven neuroinflammation in AD remain elusive.

**Methods:** The circular RNA Dlg1 (circDlg1) was identified using circRNA microarray screening in magnetic-isolated microglia of APP/PS1 mice. CircDlg1 expression in microglia of APP/PS1 mice and AD patients was validated by FISH. Flow cytometry and immunostaining were conducted to explore the roles of circDlg1 in microglia. Adeno-associated virus 9 preparations for interfering with microglial circDlg1 were microinjected into mouse lateral ventricle to explore influences on microglial response, neuroinflammation and AD pathologies. Y-maze, novel object recognition and Morris water maze tasks were performed to assess cognitive performance. RNA pulldown assays, mass spectrometry analysis, RNA immunoprecipitation, and co-immunoprecipitation were performed to validate the underlying regulatory mechanisms of circDlg1.

**Results:** A novel circular RNA circDlg1 was observed elevated using circRNA microarray screening in microglia isolated from APP/PS1 mice and validated increased in intracerebral microglia of AD patients. Microglia-specific knockdown of circDlg1 remarkably ameliorated microglial recruitment and envelopment of amyloid-β (Aβ), mitigated neuroinflammation, and prevented cognitive decline in APP/PS1 mice. Mechanistically, circDlg1 interfered with the interaction between phosphodiesterase 4b (PDE4B) and Smurf2, an E3 ubiquitin ligase of PDE4B. The formed ternary complex protected PDE4B from ubiquitination-dependent degradation via unique N-terminal targeting domain, thus consequently decreasing cAMP levels. We further confirmed that microglial circDlg1 downregulation significantly activated PKA/CREB anti-inflammatory pathway by decreasing PDE4B protein levels in APP/PS1 mice.

**Conclusion:** The novel microglia-upregulated circDlg1 tightly involves in neuroinflammation in APP/PS1 mice via determining the protein fate of PDE4B. Microglial loss of circDlg1 promotes microglial protective response to Aβ deposition and relieves neuroinflammation, thus suggesting a potential therapeutic strategy that specifically targets the microglial response in AD.

## Introduction

A wealth of evidence underscores the pivotal role of microglia in Alzheimer's disease (AD) pathogenesis, where microglial robust immune response and neuroinflammation are key drivers of AD process 1, 2. Microglia activation has been considered as an early event of AD for its early appearance even before mild cognitive impairment (MCI), preceding the formation and deposition of Aβ plaques 2-4. Abnormal microgliosis and microglial impaired response to Aβ deposition, including uptake of Aβ, recruitment and envelopment of Aβ plaques aggravate amyloid pathology 5. Inflammatory molecules released from activated microglia are notably detrimental, contributing significantly to synaptic impairment, neuronal death, and neurogenesis inhibition 6. Furthermore, advances in genetics have shed light on many immune genes subserving this microglial response to the AD susceptibility. AD risk loci such as CR1, CD33, and TREM2 exhibit pronounced or exclusive expression in microglia compared with other cells of the central nervous system (CNS) 7-9. Therefore, as the resident immune cells of CNS, microglia are the responders and contributors of AD. Although the growing focus on the complex and fascinating character of microglia 10, the precise nature of their involvement and the cellular mechanisms underlying AD are still ambiguous.

Circular RNAs (circRNAs) are endogenous noncoding RNA molecules enriched in brain cells but still mysterious in neurodegenerative disease. CircRNAs are formed by back-splicing of genes and have gained great attention due to their stability, conservation and tissue/developmental-stage-expression specificity 11, 12. Their cellular functions are quite diverse, encompassing modulating transcription of parental genes in the nucleus, sponging microRNAs (miRNAs), forming circRNA-protein/mRNA complexes, and translating proteins in the cytoplasm 13, 14. In fact, the significant associations between circRNA expression and AD severity have been uncovered 15. Our previous research, in concert with others, has revealed that circRNA levels are obviously dysregulated in the vulnerable brain region of AD patients and mouse models, pointing the importance of circRNAs in this most common neurodegenerative disease 15-17. Given that a comprehensive inventory of circRNAs has been revealed in neurons 12, most studies have put efforts into the regulatory function of these neuronal-enriched circRNAs in AD 17-19. A recent study has characterized the circRNA spectrum in non-neuronal cells as well 20. It is amazing that the amount of circRNAs in peripheral blood mononuclear white cells is equivalent to that in neurons 20, suggesting the vastly underestimated manifestation of circRNAs in microglia, the intracerebral immune cells. A latest study has revealed that microglial circ-UBE2K is tightly associated with microglia activation and immune inflammation in depression 21. However, there is not any clue of the expression profile and regulatory role of circRNAs in the microglia of AD. Therefore, characterizing the circRNA spectrum in microglia and elucidating its critical role in AD will provide a new perspective on microglia-driven neuroinflammation and AD progression.

In this study, we used magnetic-activated cell sorting (MACS) to isolate CD11b^+^ cells from the cortex of APP/PS1 (APPswe and PSEN1dE9) AD model mice, and identified a novel microglia-enriched circRNA (circDlg1), which was stably expressed and specifically upregulated in the microglia of APP/PS1 mice and AD patients. CircDlg1 knockdown in microglia remarkably enhanced microglia-mediated Aβ engagement, mitigated neuroinflammation, and thereby ameliorated synaptic impairment and cognitive deficits of APP/PS1 mice. Mechanistically, circDlg1 acted as a blocker of phosphodiesterase 4b (PDE4B) and Smurf2, an E3 ubiquitin ligase of PDE4B 22, and impeded the ubiquitination-dependent degradation of PDE4B via N-terminal targeting domain (TD), thus leading to the accumulation of PDE4B and deactivation of downstream cAMP/PKA/CREB anti-inflammation pathway in microglia. Our findings firstly identified a novel microglia-upregulated circRNA, circDlg1, that modulated microglial response associated with AD. The discovery of the hitherto unknown post-translational regulatory mechanism of PDE4B mediated by circDlg1 suggests possible strategies in the development of therapeutic compounds targeting microglial response in AD.

## Results

### CircDlg1 is a conserved and stable circRNA that is specifically up-regulated in the microglia of AD

**Figure 1:**
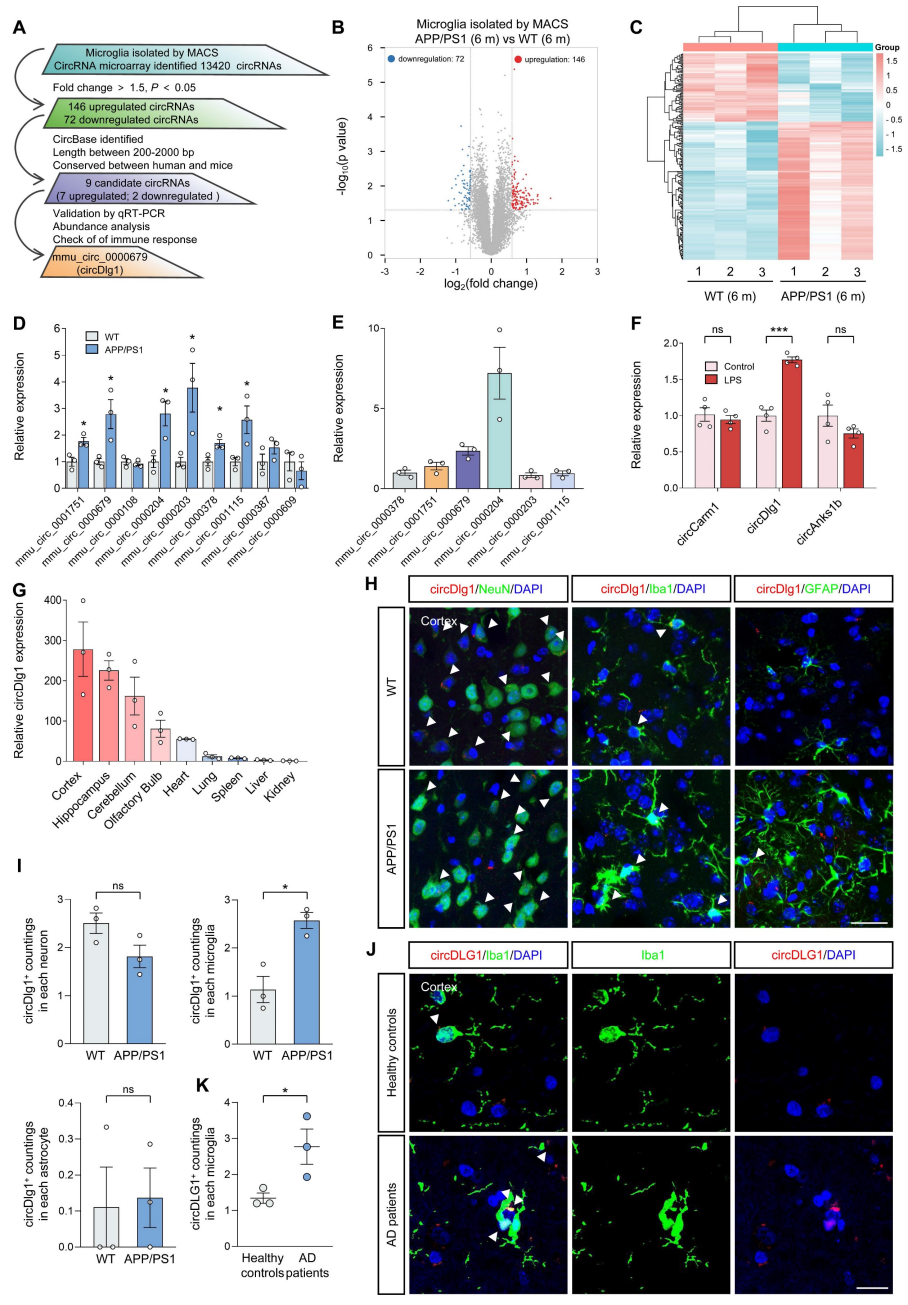
** CircDlg1 is specifically up-regulated in the microglia of AD and in LPS-treated BV-2 cells.** (A) Screening schematic of mmu_circ_0000679 (circDlg1) from cortical microglia isolated from 6-month-old male WT and APP/PS1 mice. (B) Volcano plot of downregulated (blue points), upregulated (red points), and no significant different (gray points) circRNAs in cortical microglia isolated from 6-month-old male WT and APP/PS1 mice by MACS (n = 3 mice per group). The cut-off fold change was 1.5. The cut-off *P* value was 0.05. (C) Heat map of differentially expressed circRNAs (fold change > 1.5, *P* < 0.05) (n = 3 mice per group). (D) qRT-PCR assays for the relative expression of circRNAs in cortical microglia (n = 3 mice per group). (E) qRT-PCR assays for the relative abundance of six differentially expressed circRNAs in cortical microglia (n = 3 mice per group). (F) qRT-PCR assays for the relative expression of circRNAs in BV-2 cells treated with LPS (100 ng/ml) (n = 4 biologically independent experiments). mmu_circ_0001751: circCarm1. mmu_circ_0000679: circDlg1. mmu_circ_0000204: circAnks1b. (G) qRT-PCR assays for the relative expression of circDlg1 in cortex, hippocampus, cerebellum, olfactory bulb, heart, lung, spleen, liver, and kidney. (H) FISH combined with immunostaining was performed to detect the colocalization between circDlg1 and neurons (NeuN), microglia (Iba1), and astrocytes (GFAP) in the cortex of 6-month-old male WT and APP/PS1 mice. The white triangular arrow pointed to circDlg1 and neuron/microglia/astrocyte colocalization (coloc.). Scale bar = 20 μm. (I) The average circDlg1^+^ countings per cell in (H) were shown (n = 3 mice per group). (J) FISH combined with immunostaining was performed to detect the colocalization between circDlg1 and microglia in the cortex of healthy controls and AD patients. The white triangular arrow pointed to circDlg1 and microglia coloc. Scale bar = 20 μm. (K) The average circDLG1^+^ countings per microglia in (J) were shown (n = 3 donors per group). Data were presented as mean ± SEM. Two-tailed t-tests were used. *P < 0.05, ***P < 0.001.

To investigate the role of circRNAs in the microglia of AD, we isolated cortical microglia from 6-month-old male wild-type (WT) and APP/PS1 mice by MACS using a CD11b antibody 23, 24. Cx3cr1, a microglia marker 25, was predominantly presented in CD11b^+^ cells with negligible expression in CD11b^-^ cells (Figure S1A), indicating the successful isolation. We then conducted a circRNA microarray and implemented a multi-step screening process to identify circRNAs with potential regulatory significance in microglia (Figure 1A). A total of 13420 circRNAs were detected in all chromosomes except mitochondrial chromatin (Figure S1B) and 78.79% (10573/13420) were grouped into exonic circRNAs (Figure S1C and Table S1). Among these, 218 differentially expressed circRNAs (with a fold change > 1.5, *P* < 0.05) were distributed in all chromosomes except mitochondrial chromatin and Y chromosome (Figure S1D). 146 circRNAs were upregulated while 72 circRNAs were downregulated in cortical microglia of APP/PS1 mice compared with WT mice (Figure 1B-C and Table S1). We narrowed our focus to 9 candidate circRNAs documented in circBase, with lengths between 200-2000 bp, and identified by Rybak-Wolf *et al.* as conserved between human and mouse (Figure S1E) 12. qRT-PCR results validated that 6 of these 9 circRNAs exhibited significant changes in the cortical microglia of APP/PS1 mice (Figure 1D). We then performed an abundance analysis on the 6 differentially expressed circRNAs (Figure 1E) and detected the expression of the top 3 abundant circRNAs, mmu_circ_0000204 (circAnks1b), mmu_circ_0000679 (circDlg1) and mmu_circ_0001751 (circCarm1) in lipopolysaccharide (LPS)-treated mice BV-2 cells 26, 27. Only circDlg1 was upregulated, while circAnks1b and circCarm1 remained unchanged (Figure 1F). Aβ_42_ also caused the upregulation of circDlg1 in BV-2 cells (Figure S2A). Moreover, LPS and Aβ_42_ stimulation increased the expression of has_circ_0123248 (circDLG1) in human HMC3 cells (Figure S2B). Consequently, we focused on the expression pattern and functional characterization of circDlg1 in the microglia of AD.

CircDlg1 was highly abundant in the cortex and hippocampus, two vulnerable cerebral regions in AD (Figure 1G) 28, 29. Fluorescence in situ hybridization (FISH) combined with immunostaining showed that circDlg1 was specifically elevated in cortical microglia with no significant changes in neurons or astrocytes of APP/PS1 mice (Figure 1H-I). Similar results were obtained by qRT-PCR analysis (Figure S2C). Meanwhile, microglia of AD patients expressed more circDLG1 than those of healthy controls, further demonstrating the clinical significance of circDLG1 in AD pathology (Figure 1J-K). Linear Dlg1 mRNA expression in cortical microglia of WT and APP/PS1 mice was unchanged (Figure S2D). Collectively, these findings indicate that circDlg1 is specifically elevated in the microglia of AD.

CircDlg1 (423 bp) was derived from exon 12, 13, and 14 of Dlg1 gene (Figure S3A). Amplification of circDlg1 by divergent primers in cDNA rather than gDNA and the reduced efficiency of oligo dT primers during reverse transcription both demonstrated the circular form of circDlg1 (Figure S3B-C). CircDlg1 was more resistant to the RNase R digestion and exerted greater stability under the treatment of Actinomycin D (AcD, a transcription inhibitor) than the linear transcript (Figure S3D-E). CircDlg1 was conserved between human and mouse (Figure S3F). qRT-PCR and FISH assays showed that circDlg1 was predominantly located in the cytoplasm (Figure S3G-H). Taken together, we characterize cytoplasmic circDlg1 is conserved and stable in microglia.

### CircDlg1 switches microglial polarization and knockdown of circDlg1 facilitates amyloid uptake *in vitro*

**Figure 2:**
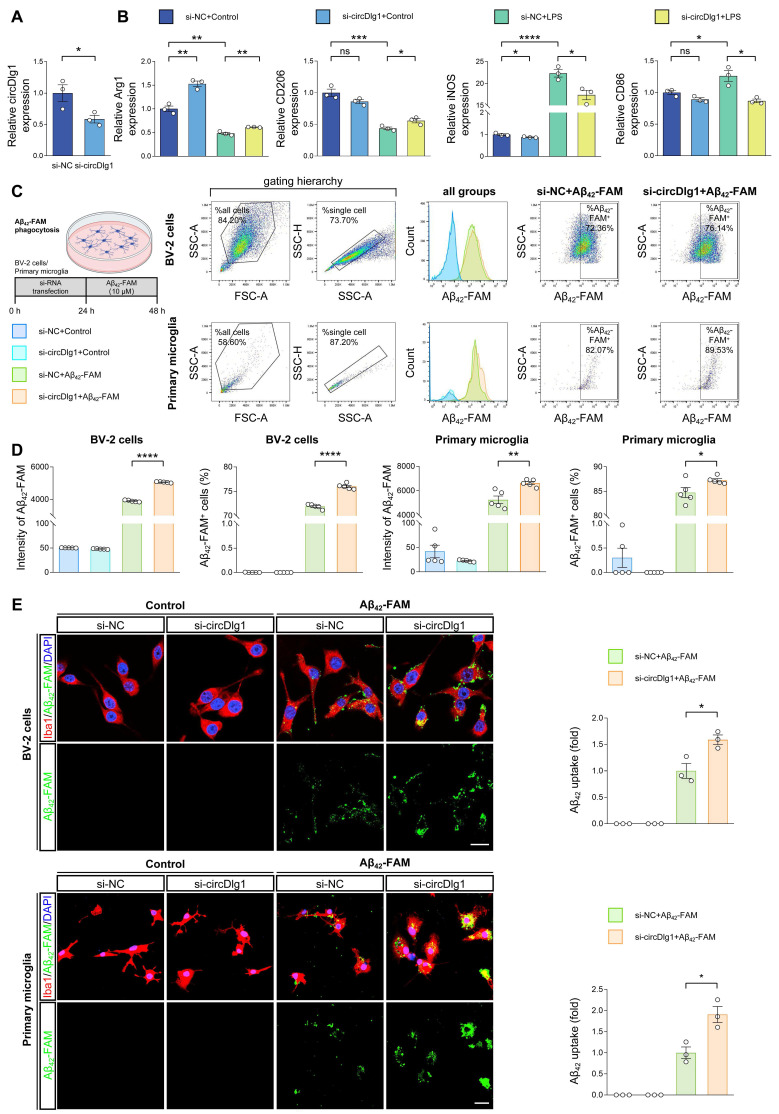
** Knockdown of circDlg1 facilitates microglial M2 polarization and amyloid uptake *in vitro.*** (A) qRT-PCR assays for the relative expression of circDlg1 in BV-2 cells transfected with si-NC or si-circDlg1 (n = 3 biologically independent experiments). (B) qRT-PCR assays for the relative expression of Arg1, CD206, iNOS, and CD86 in BV-2 cells transfected with si-NC or si-circDlg1 followed by treatment of LPS (100 ng/ml) for 18 h (n = 3 biologically independent experiments). (C-D) BV-2 cells and primary microglia transfected with si-NC or si-circDlg1 followed by treatment of Aβ_42_-FAM (10 μM) for 24 h were analyzed by flow cytometry (n = 5 biologically independent experiments). Each dot of primary microglia represented cells pooled from 6-8 neonatal brains. FSC: forward and side scatter. SSC-A: side scatter area. SSC-H: side scatter height. (C) The schematic of experiment, the sorting scheme, representative images of the number and intensity of Aβ_42_-FAM in single cells, and representative images of the percent of Aβ_42_-FAM^+^ microglia were shown. (D) Quantification of the intensity of Aβ_42_-FAM and percent of Aβ_42_-FAM^+^ microglia (n = 5 biologically independent experiments). (E) Representative images of microglia (Iba1) and Aβ_42_-FAM in BV-2 cells and primary microglia transfected with si-NC or si-circDlg1 followed by treatment of Aβ_42_-FAM (10 μM) for 24 h. Aβ_42_ uptake was quantified on the right (n = 3 biologically independent experiments). Each dot of primary microglia represented cells pooled from 6-8 neonatal brains. Scale bar = 20 μm. Data were presented as mean ± SEM. Two-tailed t-tests were used. *P < 0.05, **P < 0.01, ***P < 0.001, ****P < 0.0001.

Multiple studies have found that modulating microglial polarization by converting M1 microglia into M2 can effectively mobilize microglial protective function in AD 30-32. We then explored the role of circDlg1 in microglial polarization in BV-2 cells. Overexpression of circDlg1 significantly reduced the expression of M2 microglial markers (Arg1 and CD206), increased the levels of M1 microglial markers (iNOS and CD86), and even aggravated the degree of M1 polarization under the treatment of LPS (Figure S4A-C). However, downregulating circDlg1 obviously elevated the expression of the M2 microglial marker (Arg1), while concurrently decreased the level of M1 microglial marker (iNOS) (Figure S5A and Figure 2A-B). Under the LPS treatment, BV-2 cells showed attenuated levels of M2 microglial markers and enhanced expression of M1 microglial markers, which could be effectively reversed by circDlg1 knockdown (Figure 2B). These data indicate that circDlg1 acts as a key switch in microglial polarization and circDlg1 knockdown might motivate the protective function of microglia.

Amyloid uptake activity of microglia directly affects Aβ clearance in plaque pathology 33, 34. To investigate whether circDlg1 regulates the Aβ uptake of microglia, we conducted a flow cytometry-based assay *in vitro*. Knockdown of circDlg1 significantly facilitated Aβ_42_-FAM uptake both in BV-2 cells and primary microglia (Figure S5B and Figure 2C-D). Likewise, a significant increase in Aβ_42_-FAM phagocytosis was observed using immunostaining (Figure 2E). These data demonstrate that circDlg1 plays an important role in modulating Aβ phagocytic activity of microglia. Taken together, we propose that circDlg1 plays an essential role in regulating microglial response to Aβ deposition in AD pathology.

### Microglia-specific knockdown of circDlg1 ameliorates microglial response and neuroinflammation in APP/PS1 mice

**Figure 3:**
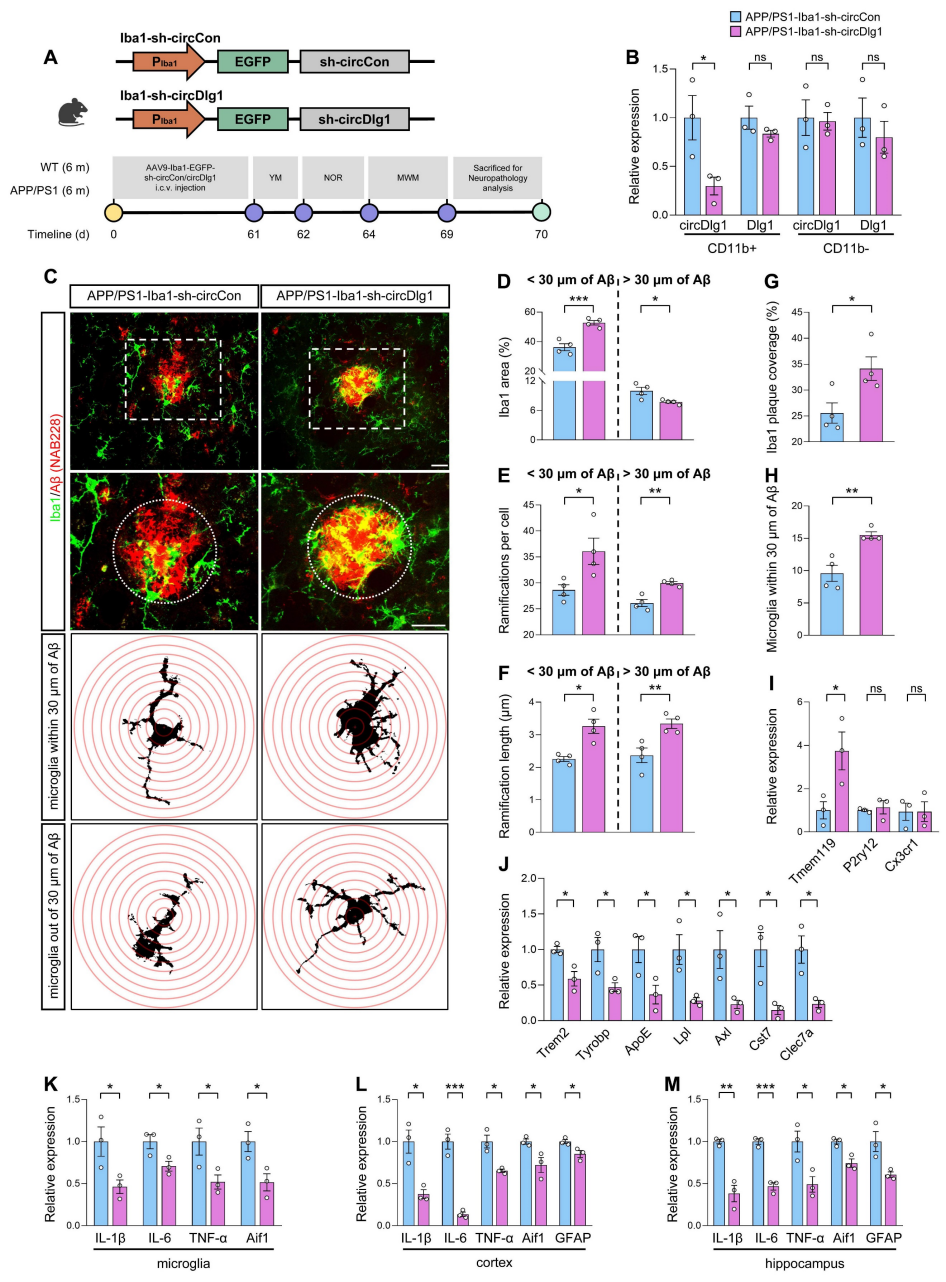
**Microglia-specific knockdown of circDlg1 ameliorates microglial response and neuroinflammation in APP/PS1 mice.** (A) Experimental schematic of 6-month-old male WT and APP/PS1 mice. 60 days after microglia-specific knockdown of circDlg1 by i.c.v.-injection, spatial learning and memory ability were examined. i.c.v.: intracerebroventricular. (B) qRT-PCR assays for the relative expression of circDlg1 and Dlg1 in CD11b^+^ and CD11b^-^ cells isolated from the brains of APP/PS1 mice injected with AAV9-Iba1-sh-circCon or AAV9-Iba1-sh-circDlg1 (n = 3 mice per group). (C) Representative images of microglia and Aβ plaque in the cortex of APP/PS1 mice injected with AAV9-Iba1-sh-circCon or AAV9-Iba1-sh-circDlg1. The dotted circle displayed the colocalization (yellow) of microglia and Aβ plaque within a radius of 30 μm, followed by skeletal analysis. Concentric circles were drawn at the center of the soma with a 0.5 μm step in Sholl analysis. Scale bar = 20 μm. (D-F) Iba1 area (D), ramifications per cell (E), ramification length within/outside 30 μm of the Aβ plaque (F) in (C) were quantified (n = 4 mice per group). (G-H) Iba1 coverage of Aβ plaque (G) and number of microglia within 30 μm of Aβ plaque (H) in (C) were quantified (n = 4 mice per group). (I-J) qRT-PCR assays for the relative expression of homeostasis- (I) and DAM-genes (J) in microglia isolated from the brains of APP/PS1 mice injected with AAV9-Iba1-sh-circCon or AAV9-Iba1-sh-circDlg1 (n = 3 mice per group). (K-M) qRT-PCR assays for the relative expression of neuroinflammation-related genes in microglia (K), cortex (L), and hippocampus (M) of APP/PS1 mice injected with AAV9-Iba1-sh-circCon or AAV9-Iba1-sh-circDlg1 (n = 3 mice per group). Data were presented as mean ± SEM. Two-tailed t-tests were used. *P < 0.05, **P < 0.01, ***P < 0.001.

Based on the results that circDlg1 regulated microglial polarization and Aβ phagocytic activity *in vitro*, we proceeded to elucidate the role of circDlg1 in microglia *in vivo*. Adeno-associated virus9 (AAV9) preparations expressing either control or circDlg1 shRNA with Enhanced Green Fluorescent Protein (EGFP) signal under Iba1 promoter (Iba1-sh-circCon/Iba1-sh-circDlg1) were microinjected into the lateral ventricle of 6-month-old male WT and APP/PS1 mice 35. We then analyzed the microglial response and related AD pathology as well as memory and spatial learning ability after 2 months (Figure 3A). EGFP signal was extensively distributed in the cortex and hippocampus and colocalized with Iba1-positive microglia (Figure S6A-B), indicating a strong microglia-specific infective efficiency. CircDlg1 was significantly reduced in APP/PS1-Iba1-sh-circDlg1 microglia compared with APP/PS1-Iba1-sh-circCon microglia (CD11b^+^ cells), with no significant reduction in CD11b^-^ cells (Figure 3B). As expected, shRNA targeting circDlg1 did not knock down linear Dlg1 levels (Figure 3B).

Microglia play a critical role in monitoring CNS parenchyma characterized by dynamic morphology changes and release of cytokines 36. Microglia in AD manifest reactive microgliosis phenotype, which is typified by low and short ramifications 37. In our study, APP/PS1-Iba1-sh-circDlg1 mice featured enhanced microglia activation in close proximity (within 30 μm) to Aβ plaques, contrasted with a diminished activation in distant regions (Figure 3C-D). We shaped microglial morphology and found that microglia after circDlg1 knockdown formed increased number of ramifications and elongated processes, akin to a surveillance state (Figure 3C and Figure 3E-F). To assess microglia-Aβ plaque interactions, we quantified microglia abundance through microglial area and number of microglia within 30 μm of Aβ plaques and observed that circDlg1 downregulation increased the recruitment and envelopment of microglia to Aβ plaques (Figure 3G-H).

Results of qRT-PCR showed a remarkable increase in the homeostatic gene (Tmem119) and significant decrease in disease-associated microglia (DAM) genes (Trem2, Tyrobp, ApoE, Lpl, Axl, Cst7, and Clec7a) in microglia isolated from APP/PS1-Iba1-sh-circDlg1 mice (Figure 3I-J). In addition, the expression of pro-inflammatory genes including IL-6, IL-1β, and TNF-α in microglia, cortex and hippocampus of APP/PS1-Iba1-sh-circDlg1 mice were significantly decreased, alongside a pronounced reduction of glial cell activation markers (Aif1 and GFAP) (Figure 3K-M). We also investigated the effects of circDlg1 knockdown in microglia of APP/PS1 mice at the age of 3 months (Figure S7A-B), a stage that preceded Aβ deposition but appeared microglia activation 38. Microglia after circDlg1 knockdown featured attenuated activation and increased ramified processes, suggesting that microglia exhibited well surveillance of brain tissue (Figure S7C-F). In addition, reduction of pro-inflammatory genes was accompanied by decreased glial cell activation markers (Figure S7G-I). These results support that microglia-specific knockdown of circDlg1 effectively deploys microglial engagement with Aβ plaques and alleviates neuroinflammatory state in APP/PS1 mice.

### Downregulation of circDlg1 in microglia alleviates AD pathologies and cognitive dysfunction in APP/PS1 mice

**Figure 4:**
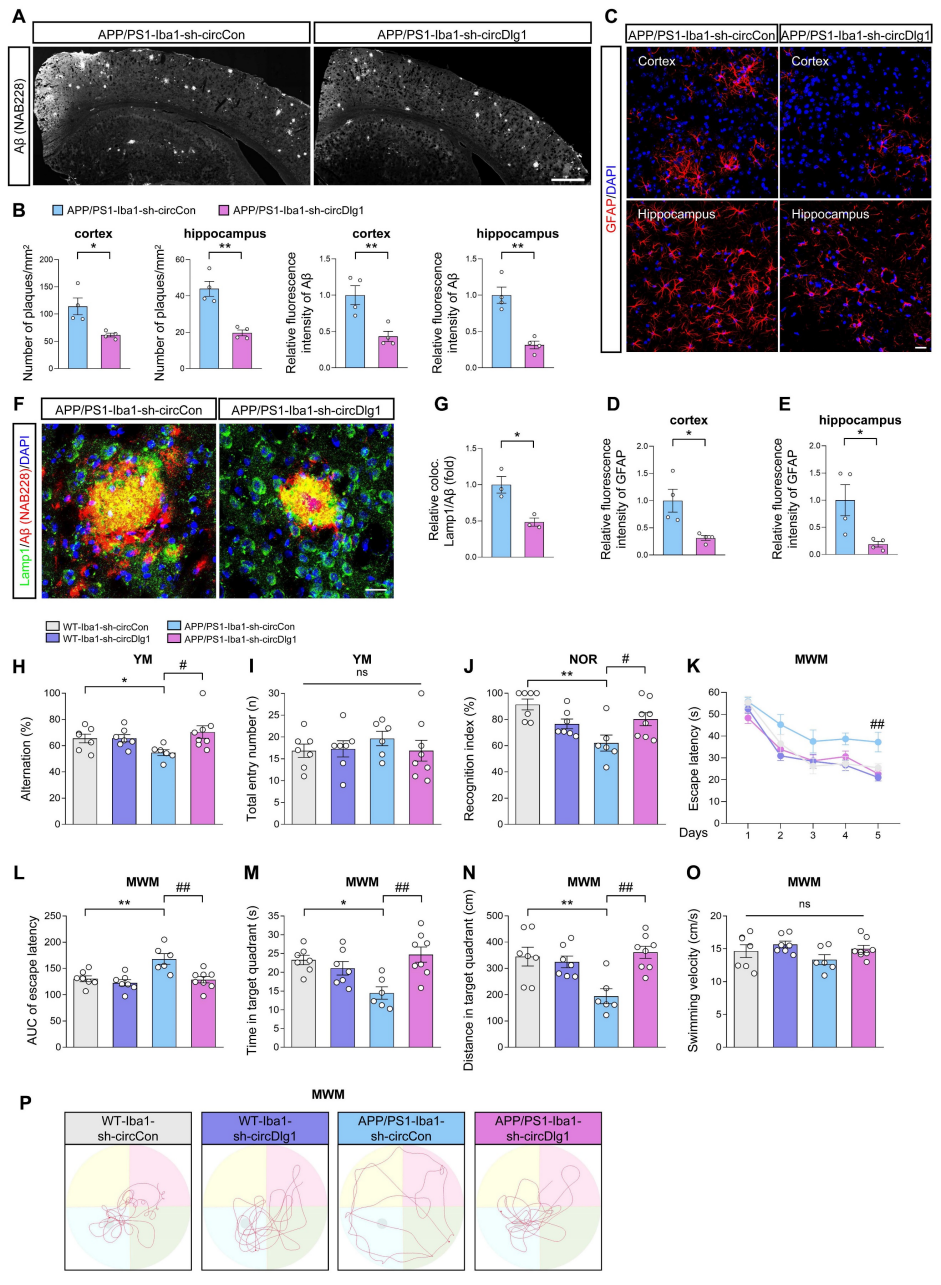
**Downregulation of circDlg1 in microglia alleviates AD pathologies and cognitive dysfunction in APP/PS1 mice.** (A) Representative images of Aβ plaques in brain sections of APP/PS1 mice injected with AAV9-Iba1-sh-circCon or AAV9-Iba1-sh-circDlg1. Scale bar = 500 μm. (B) Number of plaques/mm^2^ and relative fluorescence intensity of Aβ plaques in the cortex and hippocampus in (A) were quantified (n = 4 mice per group). (C) Representative images of astrocytes in brain sections of APP/PS1 mice injected with AAV9-Iba1-sh-circCon or AAV9-Iba1-sh-circDlg1. Scale bar = 20 μm. (D-E) Relative fluorescence intensity of GFAP in cortex (D) and hippocampus (E) in (C) was quantified. (F) Representative images of colocalization (yellow) of dystrophic neurites (Lamp1) and Aβ plaques in the cortex of APP/PS1 mice injected with AAV9-Iba1-sh-circCon or AAV9-Iba1-sh-circDlg1. Scale bar = 20 μm. (G) The relative percent of dystrophic neurites and Aβ plaque colocalization (coloc.) in (F) was quantified (n = 3 mice per group). (H-J) Working memory was assessed by Y-maze (YM) task and recognition memory was assessed by novel object recognition (NOR) task (n = 6-8 mice per group). Statistical analysis was performed by two-way ANOVA followed by Tukey's post hoc test. *P < 0.05, **P < 0.01 versus WT-Iba1-sh-circCon group; ^#^*P* < 0.05 versus APP/PS1-Iba1-sh-circCon group. The percentage of spontaneous alternations (H) and total entry numbers (I) in YM task were analyzed. Recognition index (%) (J) in NOR task was analyzed. (K-P) Spatial learning and memory was assessed by Morris water maze (MWM) task (n = 6-8 mice per group). Statistical analysis was performed by two-way ANOVA followed by Tukey's post hoc test. *P < 0.05, **P < 0.01 versus WT-Iba1-sh-circCon group; ^##^*P* < 0.01 versus APP/PS1-Iba1-sh-circCon group. (K) The escape latency to reach the hidden platform in the MWM task during the 5-day training phase. (L) The AUC of escape latency during the training phase. AUC: area under the curve. (M-N) Time spent (M) and distance covered (N) in the target quadrant. (O) The swimming velocity in the probe trial. (P) Representative swimming trajectories of each group. The gray circle represented the hidden platform. Data were presented as mean ± SEM. Two-tailed t-tests were used unless otherwise specified. *P < 0.05, **P < 0.01.

We then conducted a comprehensive neuropathological analysis to gain a deeper understanding of how microglia-specific knockdown of circDlg1 effectively affected AD-associated neuropathology. Aβ plaque burden, the typical pathology of AD, significantly relieved in the cortex and hippocampus of APP/PS1-Iba1-sh-circDlg1 mice, which coincided with the upregulation of microglial coverage over Aβ plaques (Figure 4A-B). Meanwhile, alleviated microglial activation and reduced inflammatory cytokines in APP/PS1-Iba1-sh-circDlg1 mice contributed to reduced astrocyte activation (Figure 4C-E) and diminished Lamp1^+^ dystrophic neurites (Figure 4F-G).

To investigate whether ameliorated AD-associated neuropathology after microglia-specific knockdown of circDlg1 translated to cognitive improvement, Y-maze task, novel object recognition (NOR) task, and Morris water maze (MWM) task were performed 39, 40.

In the Y-maze task, APP/PS1-Iba1-sh-circCon mice had a reduced spontaneous alternation compared with WT-Iba1-sh-circCon mice whereas microglial circDlg1 reduction in APP/PS1 mice prevented short-term memory decline (Figure 4H). There was no difference in motor function among all four groups according to the similar total entry numbers (Figure 4I). Consistently, APP/PS1-Iba1-sh-circDlg1 mice showed obviously improved recognition memory compared with the APP/PS1-Iba1-sh-circCon mice in the NOR task (Figure 4J). In the MWM task, APP/PS1-Iba1-sh-circDlg1 mice manifested an obviously shorter latency to find the hidden platform compared with APP/PS1-Iba1-sh-circCon mice in the training period, indicating improved learning and spatial memory (Figure 4K-L). During probe trials, memory retention was measured by the time spent and the distance covered in the quadrant where the hidden platform was removed. APP/PS1-Iba1-sh-circCon mice showed a tendency towards reduced memory retention compared with WT-Iba1-sh-circCon mice, which was reversed by microglial circDlg1 knockdown (Figure 4M-N). Four groups of mice had similar swimming velocity and the trajectories were shown (Figure 4O-P). These findings illustrate that microglia-specific knockdown of circDlg1 rescues cognitive decline in APP/PS1 mice.

### CircDlg1 interacts with N-terminal targeting domain (TD) of phosphodiesterase 4b (PDE4B) in microglia

To explore the molecular mechanism of circDlg1 in microglia-mediated neurodegenerative progression of AD, we conducted RNA pulldown assays to detect whether circDlg1 had the ability to sponge miRNAs 41. CircDlg1 did not appear to sequester miRNAs, as evidenced by its lack of enrichment upon Ago2 (Figure S8A). In order to analyze the coding potential of circDlg1, we utilized a Coding Potential Assessment Tool (http://lilab.research.bcm.edu/calculator_sub.php). The results showed that circDlg1 had an extremely low coding ability (coding probability = 0.35) (Figure S8B).

**Figure 5:**
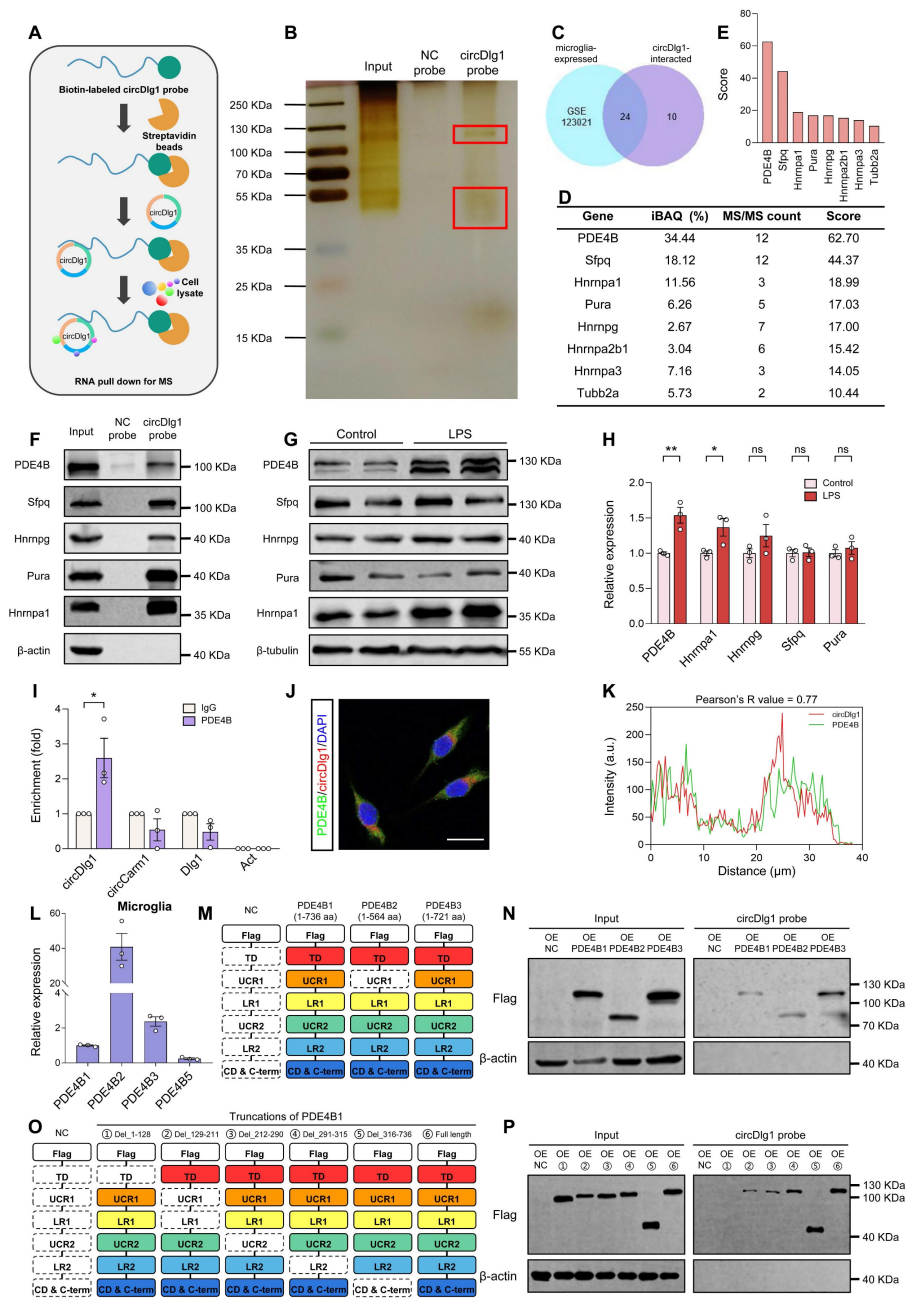
** CircDlg1 interacts with N-terminal targeting domain (TD) of phosphodiesterase 4b (PDE4B) in microglia.** (A) RNA pulldown assays were performed using biotin-labeled circDlg1 probe followed by MS. MS: mass spectrometry. (B) RNA pulldown assays combined with SDS/PAGE and silver staining were performed to detect circDlg1-protein complex in the cortex of 6-month-old male WT mice. Proteins interacting with circDlg1 in the red rectangle were identified by MS. (C) Venn diagram showed 24 proteins as microglia-related proteins in common shared in both groups, microglia single-cell RNA sequencing (GSE123021) and proteins interacting with circDlg1 identified by MS. (D) The top 5 scored proteins in iBAQ and MS/MS count (8 in total) were listed. iBAQ: intensity based absolute quantification. (E) The score of the 8 proteins listed in (D). (F) WB after RNA pulldown assays using NC or circDlg1 probe was performed to verify the interaction between circDlg1 and the top 5 proteins (PDE4B, Sfpq, Hnrnpa1, Pura, and Hnrnpg) in the cortex of 6-month-old male WT mice (n = 3 mice). (G) Protein expression of PDE4B, Sfpq, Hnrnpa1, Pura, and Hnrnpg after LPS treatment (100 ng/ml) of BV-2 cells was detected by WB. (H) Relative protein levels in (G) were quantified (n = 3 biologically independent experiments). (I) Interaction between circDlg1 and PDE4B was assessed by RNA immunoprecipitation and qRT-PCR assays in BV-2 cells transfected with PDE4B (n = 3 biologically independent experiments). IgG was used as a negative control. (J) FISH combined with immunostaining was performed to detect the colocalization between circDlg1 and PDE4B in BV-2 cells (n = 3 biologically independent experiments). (K) Fluorescence intensity profiles and Pearson's R value of circDlg1 and PDE4B in (J) were presented. (n = 3 biologically independent experiments). Scale bar = 20 μm. (L) qRT-PCR assays for the relative abundance of PDE4B variants (PDE4B1, PDE4B2, PDE4B3, and PDE4B5) in cortical microglia of 6-month-old male WT mice (n = 3 mice). (M) Domain organization of PDE4B variants (PDE4B1, PDE4B2, and PDE4B3) was displayed. (N) WB after RNA pulldown assays using circDlg1 probe was performed to verify the interaction between circDlg1 and PDE4B variants in circDlg1-overexpressed HEK293 cells (n = 3 biologically independent experiments). (O) The truncations of PDE4B1 were displayed. (P) WB after RNA pulldown assays using circDlg1 probe was performed to verify interaction between circDlg1 and WT/truncated PDE4B1 in circDlg1-overexpressed HEK293 cells (n = 3 biologically independent experiments). Data were presented as mean ± SEM. Two-tailed t-tests were used. *P < 0.05, **P < 0.01.

Given the ample evidence that circRNAs engaged in protein-protein interactions 42, 43, we then performed RNA pulldown assays followed by mass spectrometry (MS) analysis to identify the potential binding proteins of circDlg1 in the cortex of WT mice (Figure 5A-B). A total of 34 proteins were pulled down by biotinylated circDlg1 probe, and 24 of them were expressed in microglia (Figure 5C and Figure S8C). GO functional categorization revealed an enrichment of these proteins in key biological processes, including metabolic process, signaling, response to stimulus, and immune system process (Figure S8D). A subsequent disease network analysis utilizing the Metascape database (metascape.org) underscored the involvement of these 24 proteins in most diseases accompanied by microglia-mediated neuroinflammation, reinforcing the critical role of circDlg1 in microglia during disease progression (Figure S8E).

According to intensity based absolute quantification (iBAQ) and MS/MS count, the top 5 proteins in iBAQ and MS/MS count (8 in total) were respectively scored and listed (Figure 5D-E). CircDlg1 was validated to interact with the top scored 5 proteins (PDE4B, Sfpq, Hnrnpa1, Pura, and Hnrnpg) (Figure 5F). Notably, PDE4B stood out to be the most significantly upregulated protein in LPS-activated BV-2 cells (Figure 5G-H). RIP assays also demonstrated that circDlg1, but not circCarm1 or linear Dlg1 was pulled down by PDE4B (Figure 5I), further supporting their interaction. Meanwhile, FISH combined with immunostaining assays found that circDlg1 colocalized with PDE4B (Pearson's R-value = 0.77) in the cytoplasm of BV-2 cells (Figure 5J-K).

Among variants of PDE4B, PDE4B1, PDE4B2, PDE4B3, and PDE4B5 are conserved between human and mouse 44, 45. PDE4B2 was sensitive to inflammatory stimuli and closely related with inflammatory factor and chemokine expression 46, 47. Microglial PDE4B2 initiated an inflammatory gene expression program that led to immunophenotypically activated microglia 47. Consistently, we found PDE4B2 had the highest abundance in cortical microglia of 6-month-old WT mice followed by PDE4B3, PDE4B1 and PDE4B5 (Figure 5L). Conserved PDE4B1 and PDE4B2 isoforms could be detected in BV-2 cells, with PDE4B3 and PDE4B5 showing minimal expression (Figure S9A). RNA pulldown assays showed that circDlg1 had a strong binding capability to PDE4B1, PDE4B2, PDE4B3 in HEK293 cells transfected with circDlg1 and PDE4B plasmids (Figure 5M-N). Since PDE4B1 was a long-form variant containing all functional domains of PDE4B 48, flag-labeled full-length and truncated PDE4B1 plasmids were constructed. Results of RNA pulldown assays showed that N-terminal targeting domain (TD), but not other domains, directly bound to circDlg1 (Figure 5O-P). Our results collectively illustrate that circDlg1 binds to the N-terminal TD of PDE4B in microglia.

### Microglia-specific knockdown of PDE4B limits the extent of neuroinflammation and alleviates AD pathology

PDE4B has been widely acknowledged for a major cAMP-metabolizing enzyme 49, primarily associated with modulation of inflammatory responses in immune cells including microglia 50, 51. PDE4B drives inflammatory response to spinal cord injury 52, lung injury 53, and CNS inflammation 54, 55. However, the exact role of PDE4B in microglia of AD is still ambiguous. We detected the alteration of PDE4B in APP/PS1 mice and found that PDE4B protein levels were increased in the cortex of APP/PS1 mice, but not in the hippocampus (Figure S9B-C). The mRNA levels of PDE4B were unchanged in both brain regions (Figure S9D). Immunostaining assays further verified the increased expression of PDE4B in cortical microglia of APP/PS1 mice (Figure S9E-F).

**Figure 6:**
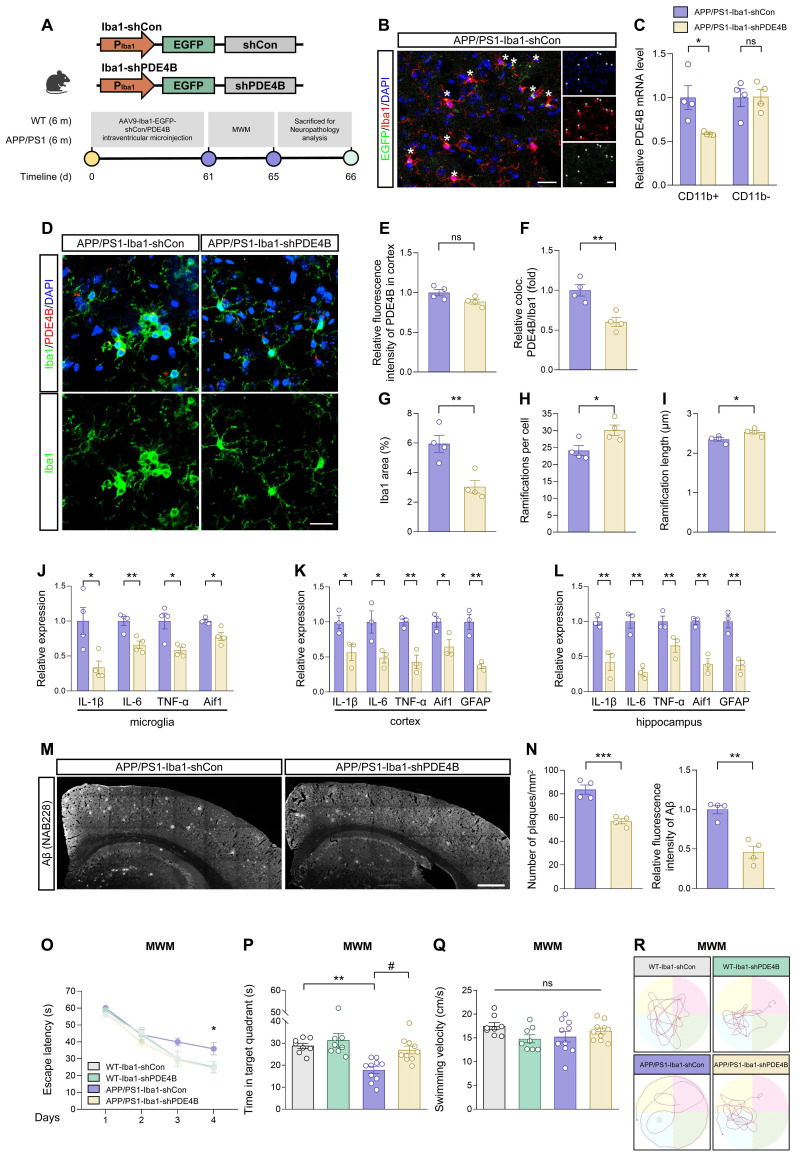
**Microglia-specific knockdown of PDE4B limits the extent of neuroinflammation and alleviates AD pathology.** (A) Experimental schematic of 6-month-old male WT and APP/PS1 mice. 60 days after microglia-specific knockdown of PDE4B by i.c.v.-injection, MWM was performed to detect spatial learning and memory ability. (B) Immunostaining was performed to detect the colocalization between AAV9 viral (EGFP) and microglia in the cortex of injected APP/PS1 mice. Scale bar = 20 μm. (C) qRT-PCR assays for the relative expression of PDE4B in CD11b^+^ and CD11b^-^ cells isolated from the brains of APP/PS1 mice injected with AAV9-Iba1-shCon or AAV9-Iba1-shPDE4B (n = 4 mice per group). (D) Representative cortical images of PDE4B and microglia colocalization (yellow) in the cortex of APP/PS1 mice injected with AAV9-Iba1-shCon or AAV9-Iba1-shPDE4B. Scale bar = 20 μm. (E-F) Relative fluorescence intensity of PDE4B in cortex (E) and the relative fold change of PDE4B and microglia coloc. (F) in (D) were quantified (n = 4 mice per group). (G-I) Total Iba1 area in cortex (G) and skeletal analysis of microglia including ramifications per cell (H) and each ramification length (I) in (D) were quantified (n = 4 mice per group). (J-L) qRT-PCR assays for the relative expression of neuroinflammation-related genes in microglia (J), cortex (K), and hippocampus (L) of APP/PS1 mice injected with AAV9-Iba1-shCon or AAV9-Iba1-shPDE4B (n = 3-4 mice per group). (M) Representative images of Aβ plaques in the brain sections of APP/PS1 mice injected with AAV9-Iba1-shCon or AAV9-Iba1-shPDE4B. Scale bar = 500 μm. (N) Number of plaques/mm^2^ and relative fluorescence intensity of Aβ in (M) were quantified (n = 4 mice per group). (O-R) Spatial learning and memory were assessed by MWM task (n = 8-10 mice per group). Statistical analysis was performed by two-way ANOVA followed by Tukey's post hoc test. (O) The escape latency to reach the hidden platform in the MWM test during the 4-day training phase. *P < 0.05 APP/PS1-Iba1-shPDE4B group versus APP/PS1-Iba1-shCon group. (P) Time spent in the target quadrant in the probe trial. **P < 0.01 versus WT-Iba1-shCon group; #*P* < 0.05 versus APP/PS1-Iba1-shCon group. (Q) The swimming velocity in the probe trial. (R) Representative swimming trajectories of each group. The gray circle represented the hidden platform. Data were presented as mean ± SEM. Two-tailed t-tests were used unless otherwise specified. *P < 0.05, **P < 0.01, ***P < 0.001.

We then explored whether microglia-specific knockdown of PDE4B affected microglial activation in APP/PS1 mice. AAV9 preparations expressing either control or PDE4B shRNA with EGFP signal under Iba1 promoter (Iba1-shCon/Iba1-shPDE4B) were microinjected into the lateral ventricle of 6-month-old male WT and APP/PS1 mice and the memory and spatial learning ability was analyzed after 2 months (Figure 6A). The colocalization of EGFP signal with Iba1-positive microglia and the reduction of PDE4B mRNA in CD11b^+^ cells indicated an effective transfection (Figure 6B-C). Immunostaining assays displayed that microglial PDE4B downregulation did not influence total PDE4B protein levels in the cortex (Figure 6D-E) but reduced PDE4B protein levels in microglia (Figure 6D and Figure 6F). We then examined the activation of microglia by morphology analysis. Microglia of APP/PS1-Iba1-shCon mice showed abnormal activation, with demounting reactive microgliosis and less and shorter ramifications, while these phenotypes were obviously ameliorated in APP/PS1-Iba1-shPDE4B mice (Figure 6D and Figure 6G-I). Furthermore, qRT-PCR assays showed obviously decreased expression of neuroinflammation-related genes after microglia-specific knockdown of PDE4B (Figure 6J-L). In addition, PDE4B knockdown increased cAMP concentration and reversed LPS-induced decline of PKA and CREB phosphorylation *in vitro* (Figure S10A-F), which curtailed the inflammatory state of microglia 56-58.

Multiple studies have reported that microglia-mediated neuroinflammation promotes the production and seeding of Aβ plaques 59, 60. We investigated whether microglia-specific knockdown of PDE4B affected Aβ pathology in APP/PS1 mice and found a significant reduction of Aβ plaque deposition in APP/PS1-Iba1-shPDE4B mice (Figure 6M-N). Moreover, MWM task was performed to detect spatial learning and memory abilities. In comparison with APP/PS1-Iba1-shCon mice, APP/PS1-Iba1-shPDE4B mice displayed significantly alleviated spatial learning memory deficits, which were manifested as a shorter latency to find the hidden platform during the 4-day training phase and more time spent in the target quadrant during the probe trial (Figure 6O-P). Of note, no obvious difference in the swimming velocity was observed among mice and the trajectories were shown (Figure 6Q-R). Collectively, these data demonstrate that downregulation of PDE4B in microglia relieves neuroinflammation, reduces Aβ burden, and rescues spatial learning and memory deficits in APP/PS1 mice.

### CircDlg1 protects PDE4B from ubiquitination-dependent degradation

**Figure 7:**
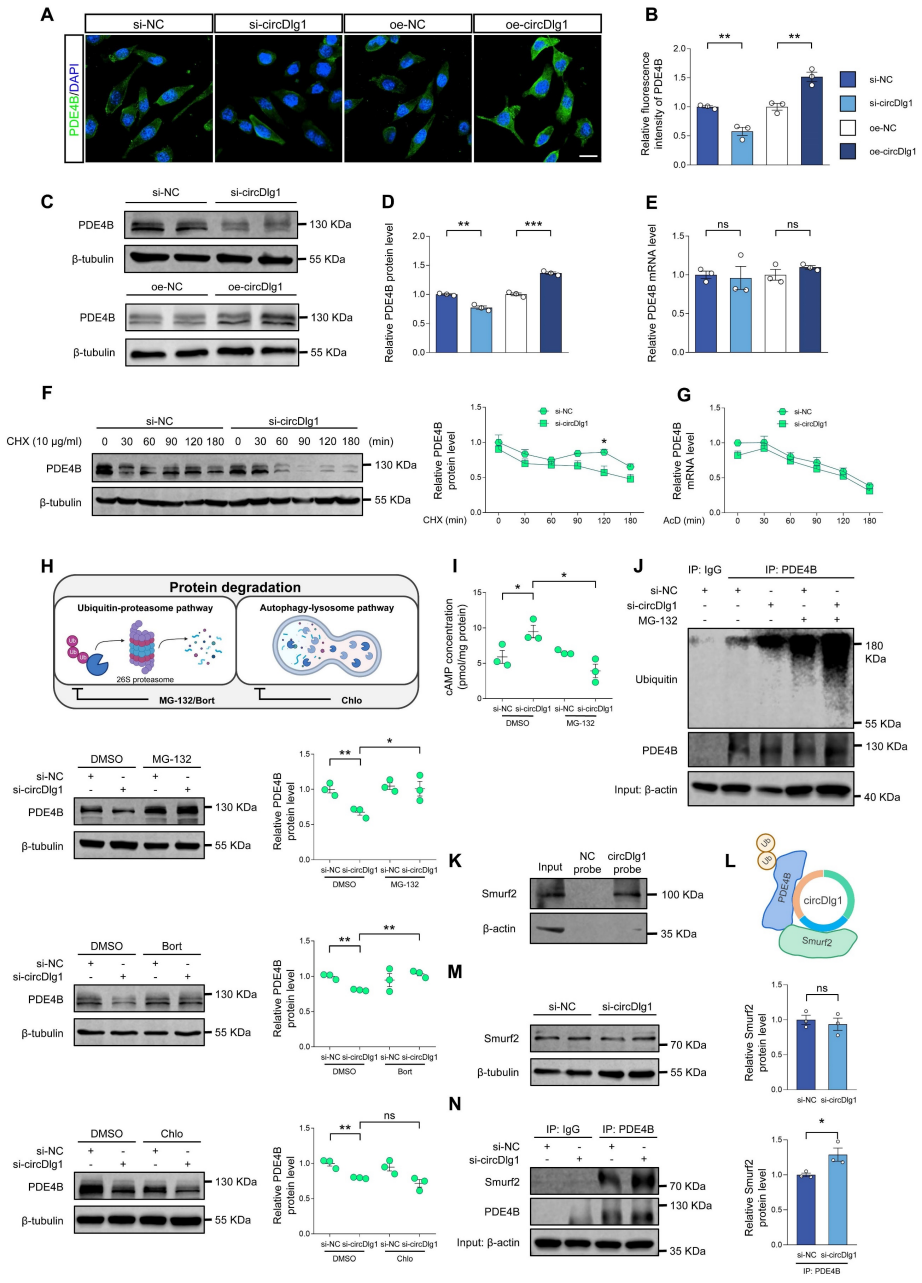
**CircDlg1 protects PDE4B from ubiquitination‑dependent degradation.** (A) Representative images of PDE4B in BV-2 cells transfected with si-NC, si-circDlg1, oe-NC, and oe-circDlg1. Scale bar = 20 μm. (B) Relative fluorescence intensity of PDE4B in (A) was quantified (n = 3 biologically independent experiments). (C) Protein expression of PDE4B in BV-2 cells transfected with si-NC, si-circDlg1, oe-NC, and oe-circDlg1 was detected by WB (n = 3 biologically independent experiments). (D) Relative PDE4B protein levels in (C) were quantified (n = 3 biologically independent experiments). (E) qRT-PCR assays for the relative expression of PDE4B in BV-2 cells transfected with si-NC, si-circDlg1, oe-NC, and oe-circDlg1 (n = 3 biologically independent experiments). (F) Protein expression of PDE4B in BV-2 cells transfected with si-NC or si-circDlg1 followed by treatment of CHX (10 μg/ml) at the indicated time points was detected by WB. Relative PDE4B protein levels were quantified on the right (n = 3 biologically independent experiments). CHX: cycloheximide. Statistical analysis was performed by two-way ANOVA followed by Tukey's post hoc test. *P < 0.05 versus si-NC group. (G) qRT-PCR assays for the relative expression of PDE4B in BV-2 cells transfected with si-NC or si-circDlg1 followed by treatment of AcD (2 μg/mL) at the indicated time points (n = 4 biologically independent experiments). Statistical analysis was performed by two-way ANOVA followed by Tukey's post hoc test. (H) Schematic diagrams showed inhibition of protein degradation by indicated inhibitors. Protein expression of PDE4B in BV-2 cells transfected with si-NC or si-circDlg1 followed by treatment of MG-132 (10 μM), Bort (200 nM) or Chlo (10 μM) for 1 h was detected by WB. Relative PDE4B protein levels were quantified on the right (n = 3 biologically independent experiments). Bort: Bortezomib. Chlo: Chloroquine. (I) ELISA detected cAMP concentration in BV-2 cells transfected with si-NC or si-circDlg1 followed by treatment of MG-132 (10 μM) for 1 h (n = 3 biologically independent experiments). (J) Immunoprecipitation detected ubiquitination of PDE4B in BV-2 cells transfected with si-NC or si-circDlg1 followed by treatment of MG-132 (10 μM) for 1 h (n = 3 biologically independent experiments). IgG was used as a negative control. Ub: ubiquitin. (K) WB after RNA pulldown assays using NC or circDlg1 probe was performed to verify interaction between circDlg1 and Smurf2 in the cortex of 6-month-old male WT mice (n = 3 mice). (L) The organization of the circDlg1-PDE4B-Smurf2 ternary complex. (M) Protein expression of Smurf2 in BV-2 cells transfected with si-NC or si-circDlg1 was detected by WB. The relative Smurf2 protein level was quantified on the right (n = 3 biologically independent experiments). (N) Immunoprecipitation detected interaction between PDE4B and Smurf2 in BV-2 cells transfected with si-NC or si-circDlg1 (n = 3 biologically independent experiments). IgG was used as a negative control. Data were presented as mean ± SEM. Two-tailed t-tests were used unless otherwise specified. *P < 0.05, **P < 0.01, ***P < 0.001.

Studies have reported that unique N-terminal TD of PDE4 isoforms involves in post-translational modifications 61-63. Given circDlg1 interaction with PDE4B via N-terminal TD and the critical role of PDE4B in the microglia of APP/PS1 mice, we dig deeply into the regulation mode of circDlg1 on PDE4B. Our results revealed that in microglia, circDlg1 knockdown reduced protein levels of PDE4B, while circDlg1 overexpression increased protein levels of PDE4B (Figure 7A-D and Figure S11A-D). However, circDlg1 did not impact mRNA levels of PDE4B (Figure 7E and Figure S11E), pointing that there was a post-translational mechanism modulating the expression of inflammatory factors in microglia (Figure S11F). An accelerated PDE4B protein degradation rate in BV-2 cells treated with circDlg1 siRNA was found under the treatment of protein synthesis inhibitor cycloheximide (CHX) (Figure 7F) and circDlg1 siRNA had no effect on levels of PDE4B mRNA under the treatment of transcription inhibitor AcD (Figure 7G), indicating that circDlg1 controlled PDE4B protein stability.

We then explored whether circDlg1 regulated PDE4B via the protein degradation pathway. Ubiquitin-proteasome pathway and autophagy-lysosome pathway are recognized as two principal mechanisms of protein degradation (Figure 7H) 64, 65. The effect of circDlg1 knockdown to decrease PDE4B protein levels could be reversed by proteasome inhibitors MG-132 and bortezomib, but not the lysosomal inhibitor chloroquine (Figure 7H), suggesting that circDlg1 regulated PDE4B degradation primarily through ubiquitin-proteasome pathway. Consistently, the intracellular cAMP levels were observed to fluctuate inversely relative to PDE4B protein levels (Figure 7H-I) 66. Furthermore, circDlg1 knockdown significantly increased the ubiquitination of PDE4B (Figure 7J). Smurf2, an E3 ubiquitin ligase, has been previously reported to facilitate ubiquitin-dependent degradation of PDE4B, but not other members of PDE4 subfamilies 22. RNA pulldown assays validated the interaction between circDlg1 and Smurf2 (Figure 7K). Therefore, circDlg1, PDE4B, and Smurf2 formed a ternary complex (Figure 7L). Interestingly, knockdown of circDlg1 did not change protein levels of Smurf2 (Figure 7M), but did increase the interaction between PDE4B and Smurf2 (Figure 7N). Taken together, these results indicate that the circDlg1-PDE4B-Smurf2 ternary complex blocks the ubiquitination-dependent degradation of PDE4B.

### Microglia-specific knockdown of circDlg1 in APP/PS1 mice activates PKA/CREB anti-inflammatory signaling pathway by downregulating PDE4B

**Figure 8:**
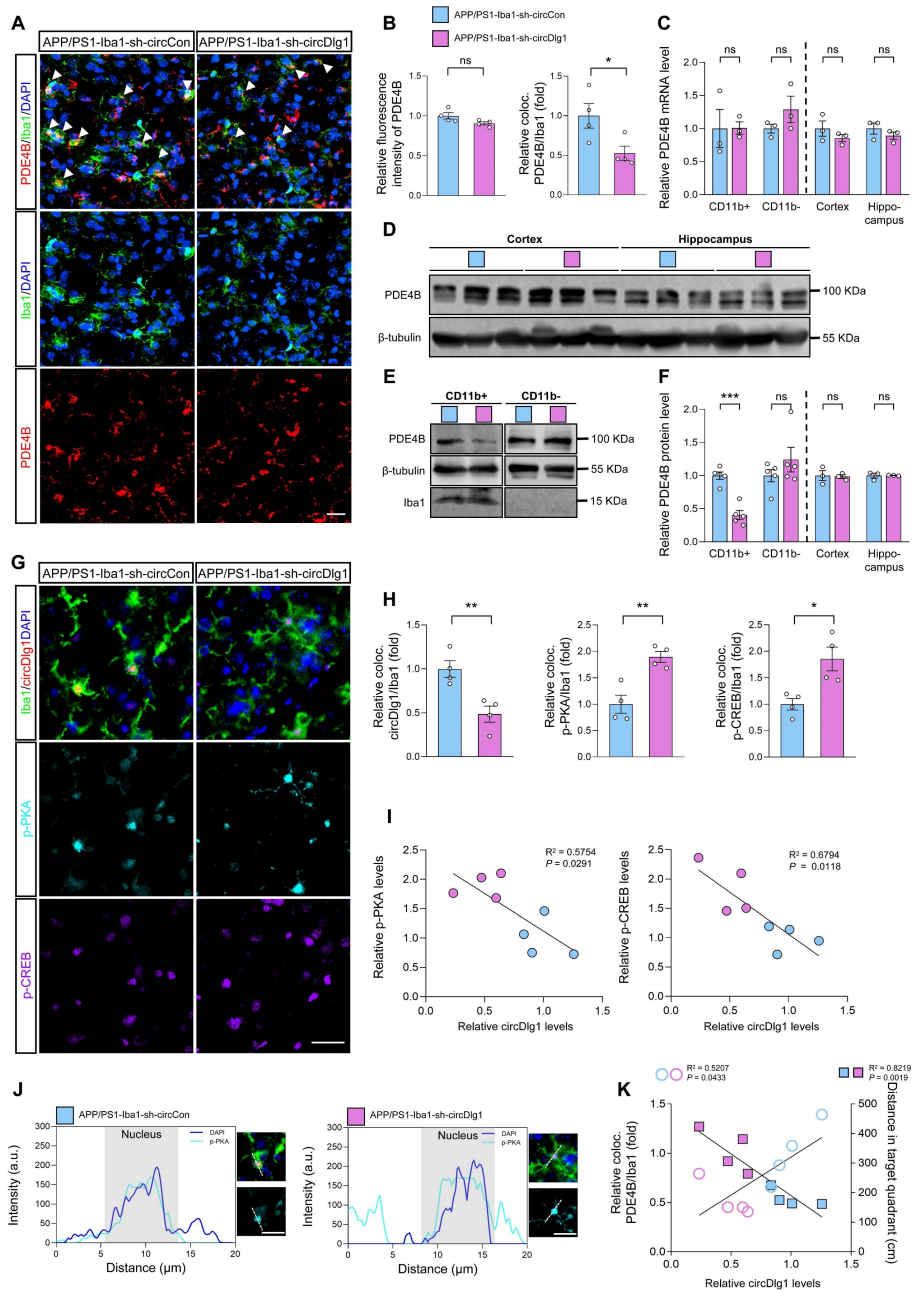
** Microglia-specific knockdown of circDlg1 in APP/PS1 mice activates PKA/CREB anti-inflammatory signaling pathway by downregulating PDE4B.** (A) Representative cortical images of PDE4B and microglia colocalization (coloc.) (yellow) in the cortex of APP/PS1 mice injected with AAV9-Iba1-sh-circCon or AAV9-Iba1-sh-circDlg1. The white triangular arrow pointed to PDE4B and microglia coloc. Scale bar = 20 μm. (B) Relative fluorescence intensity of PDE4B in cortex and the relative fold change of PDE4B and microglia coloc. in (A) were quantified (n = 4 mice per group). (C) qRT-PCR assays for the relative expression of PDE4B in CD11b^+^ cells, CD11b^-^ cells, cortex, and hippocampus of APP/PS1 mice injected with AAV9-Iba1-sh-circCon or AAV9-Iba1-sh-circDlg1 (n = 3 mice per group). (D) Protein expression of PDE4B in the cortex and hippocampus of APP/PS1 mice injected with AAV9-Iba1-sh-circCon or AAV9-Iba1-sh-circDlg1 was detected by WB (n = 3 mice per group). (E) Protein expression of PDE4B in the CD11b^+^ cells (microglia) and CD11b^-^ cells of APP/PS1 mice injected with AAV9-Iba1-sh-circCon or AAV9-Iba1-sh-circDlg1 was detected by WB (n = 5 mice per group). (F) Relative PDE4B protein levels in (D) and (E) were quantified (n = 3/5 mice per group). (G) Representative cortical images of circDlg1 and microglia colocalization (yellow), p-PKA, and p-CREB using FISH combined with TSA in the cortex of APP/PS1 mice injected with AAV9-Iba1-sh-circCon or AAV9-Iba1-sh-circDlg1. Scale bar = 20 μm. (H) The relative fold changes of microglia and circDlg1, p-PKA, and p-CREB coloc. in (G) were quantified (n = 4 mice per group). (I) Scatter plots of p-PKA/p-CREB versus circDlg1 levels in (H) were shown (n = 4 mice per group). (J) Fluorescence intensity profiles of DAPI and p-PKA in microglia were presented. Scale bar = 20 μm. (K) Scatter plots of microglial PDE4B levels detected in (B) and memory retention of MWM in Figure 4N versus microglial circDlg1 levels detected in (H) were shown (n = 4 mice per group). Data were analyzed with a linear regression method. Data were presented as mean ± SEM. Two-tailed t-tests were used unless otherwise specified. *P < 0.05, **P < 0.01, ***P < 0.001.

Considered that microglial PDE4B knockdown activated PKA/CREB anti-inflammatory pathway and circDlg1 regulated PDE4B protein levels, we validated the circDlg1-PDE4B modulatory signaling pathway *in vivo*. Immunostaining assays displayed that downregulation of circDlg1 in microglia did not influence total PDE4B fluorescence intensity in the cortex, but reduced PDE4B intensity in microglia (Figure 8A-B). The mRNA levels of PDE4B in CD11b^+^ cells, CD11b^-^ cells, cortex, and hippocampus were kept unchanged (Figure 8C). Western blot results further validated decreased PDE4B protein level only in CD11b^+^ microglia in APP/PS1-Iba1-sh-circDlg1 mice (Figure 8D-F). Microglia-specific knockdown of circDlg1 in APP/PS1 mice led to increased protein levels of the catalytic subunit of PKA (PKA α/β/γ), p-PKA and phosphorylated CREB, p-CREB in microglia (Figure 8G-H), indicating the activation of PKA/CREB anti-inflammatory signaling pathway, the key downstream pathway of PDE4B. Furthermore, we observed that the expression of circDlg1 was negatively correlated with p-PKA and p-CREB (Figure 8I). Microglial circDlg1 knockdown contributed to more p-PKA distributed both in the cytoplasm and nucleus (Figure 8J). Meanwhile, circDlg1 levels were positively correlated with PDE4B levels and negatively correlated with memory retention in APP/PS1 mice (Figure 8K). Therefore, microglial circDlg1 downregulation reduces protein levels of PDE4B and thus activates the PKA/CREB anti-inflammatory signaling pathway to ameliorate AD neuropathology in APP/PS1 mice.

## Discussion

Microglial detrimental immune response and neuroinflammation are key drivers of AD pathogenesis 13. It is of great significance to find key molecules that facilitate protective roles of microglia and prevent pro-inflammatory gene programs in microglia-driven neuroinflammation of AD. Accumulating evidence suggests that microglial circRNAs are engaged in neuropsychiatric diseases but specific characters and underlying mechanisms of circRNAs in AD remain ambiguous and need to be explored. In the present study, we unveiled a novel conserved circular RNA, circDlg1, which exhibited a distinct and abnormal upregulation specifically in the microglia of AD. Furthermore, we rigorously validated the pivotal function and regulatory mechanism of circDlg1 in microglial response to Aβ and neuroinflammation. Specifically, circDlg1 engaged in a molecular interplay with both PDE4B and Smurf2 (an E3 ubiquitin ligase of PDE4B), effectively thwarting ubiquitination-dependent degradation of PDE4B mediated by Smurf2. The aberrant accumulation of PDE4B subsequently led to an excessive breakdown of cAMP, inhibition of PKA/CREB anti-inflammatory signaling pathway, and ultimately abnormal activation of microglia (Figure S12). Downregulation of circDlg1/PDE4B axis in microglia remarkably ameliorated microglial response, neuroinflammation, Aβ pathology, and memory deficits in APP/PS1 mice. This study for the first time revealed the association between circRNA and microglia-driven AD pathogenesis, uncovered the essential role of circDlg1/PDE4B axis in microglia, and indicated the scaffold role of circDlg1 in Smurf2-mediated ubiquitination of PDE4B by interacting with the unique N-terminal TD of PDE4B, thus providing new insight into the development of innovative therapeutic strategies tailored to circDlg1-involved degradation of PDE4B for ameliorating microglial response to Aβ and neuroinflammation in AD.

Previous studies have indicated that circRNAs are more enriched in neural tissues in comparison with other tissues 12, 67, attracting global attention to delineate expression patterns and biological functions of circRNAs in neurodegenerative diseases including AD 16, 68. Despite their relatively low abundance compared to protein-coding mRNAs, circRNAs are characterized by a distinct cell type- and developmental stage-specific expression profile 12, 69. Study has found that circRNAs are highly enriched in brain and actively expressed in diversity, likely contributing to diversity and performance of brain cells 70. Our team has previously contributed to this field by identifying circCwc27, a neuron-specific circRNA, that regulates the expression of a series of AD genes via modulating Pur-α activity. Moreover, N6-methyladenosine-modified circRIMS2 has been illustrated to connect circRNA dysregulation with synaptic impairment in AD mice 19. Given the active role of microglia in Aβ pathology and synaptic homeostasis and considerable amount of circRNAs in non-neuronal cells 20, we further focus on exploring the relationship between dysregulated circRNAs in microglia and AD. It is with great interest that we have identified a conserved and stable circRNA, circDlg1, that is cell-specific upregulated in AD microglia and controls AD neuropathology by regulating microglial response.

Increasing evidence demonstrates that circRNAs have important regulatory functions by acting as miRNA sponges, forming complexes with proteins or mRNAs, or encoding small peptides 71-73. In our research, we ruled out the miRNA sponge activity of circDlg1 due to its weak interaction with Ago2, an essential protein for circRNAs to sponge miRNAs 74. Moreover, according to the online Coding Potential Assessment Tool, the coding potential of circDlg1 was predicted to be negligible. Current advancements in studies of circRNA-protein interactions have revealed their multifaceted roles as protein decoys 72, scaffolds 75, and recruiters 76, thereby affecting protein functions. In our study, we illustrated a novel regulatory mode of circRNA involved in protein-protein interactions. CircDlg1 functioned as a scaffold in the circDlg1-PDE4B-Smurf2 ternary complex, impeding the interaction between PDE4B and Smurf2. In fact, the regulatory functions of circRNAs vary when they act as protein scaffolds. For instance, circFoxo3-p21-CDK2 ternary complex enhances the interaction of CDK2 with p21 77, while circCcnb1 dissociates the formation of Ccnb1-Cdk1 complex 78. In this study, we firstly detected expression of PDE4B isoforms conserved in human and mouse (PDE4B1, PDE4B2, PDE4B3, and PDE4B5) and noted the relatively low expression of PDE4B5 in microglia. CircDlg1 interacted with long PDE4B isoforms (PDE4B1 and PDE4B3) as well as the short PDE4B2 isoform, indicating that the interaction is not specific to one particular isoform but the shared domain. Indeed, the absence of the N-terminal TD abolished the interaction between circDlg1 and PDE4B1, suggesting that the binding activity of PDE4B relied on the N-terminal TD. We further found that circDlg1-PDE4B binding through the N-terminal TD influenced the expression of PDE4B protein, rather than transcriptional expression or catalytic activity of PDE4B towards cAMP. We then demonstrated that circDlg1 enhanced the stability of PDE4B by protecting it from degradation mediated by ubiquitin-proteasomal pathway instead of the autophagy-lysosomal pathway, which was resulted from the scaffold role of circDlg1 for PDE4B and Smurf2. As a matter of fact, the N-terminal TD of PDE varies among families, subfamilies, and isoforms with pivotal modulations of subcellular location and post-translational modifications 61-63. Therefore, the regulation of PDE4B ubiquitination-dependent degradation via the binding activity of N-terminal TD with circDlg1 provides new sight into the development of strategies specifically targeting PDE4B.

Previous studies have characterized PDE4B as a key regulator of immune response in peripheral inflammatory cells, including leukocytes, bronchoalveolar monocytes/macrophages, and peritoneal macrophages 79, 80. In CNS, PDE4B, as a major enzyme that degrades cAMP in microglia, is recognized for its critical role as an immunomodulatory molecule in the microglial response to neuroinflammation 51, 81. As a matter of fact, PDE4B has been considered as an effective target for AD treatment. A large-scale genome-wide cross-trait (GWAS) identified PDE4B as a significant susceptibility locus shared between AD and gastroesophageal reflux disease 82. The application of PDE4 inhibitors reversed learning and memory deficits of APP/PS1 mice via PDE4B/PDE4D-mediated cAMP signal 83, 84. A latest study underscored the protective effect of PDE4B specific inhibition in an animal model of AD 54. Here, we conducted the microglia-specific knockdown of PDE4B in APP/PS1 mice and firstly elaborated the prominent immunomodulatory role of PDE4B in microglial function. Downregulation of PDE4B in microglia by microglia-specific knockdown of circDlg1 or PDE4B not only improved the microglial immune surveillance, but also reduced pro-inflammatory cytokines expression involved in AD pathology. By facilitating the expression of cAMP, an important molecule that controls microglial motility and morphology, PDE4B downregulation in microglia effectively restored cAMP/PKA/CREB cascade against microglia activation and recovered the protective role of microglia by driving filopodia formation. Collectively, our findings highlight the vital role of circDlg1/PDE4B regulation in microglia-driven neuroinflammation in AD.

Taken together, our data firstly uncover an abnormally upregulated circRNA, circDlg1 in the microglia of both AD patients and APP/PS1 mice and subsequently emphasize that microglia-specific knockdown of circDlg1 or the downstream effector molecule PDE4B is sufficient to maintain microglial protective response, restrain the pro-inflammatory gene program, and mitigate neuroinflammation of AD mice, thus pushing the frontier understanding of cell-specific regulation by circRNA in the microglia of AD. Furthermore, the modulation of post-translational ubiquitination of PDE4B at the N-terminal TD by circDlg1 will be generally useful for guiding more precise and safe molecular strategies for PDE4B inhibition in AD.

## Methods

### Human brain samples

The Human brain tissues for research purposes were provided by National Human Brain Bank (NHBB) for Development and Function, Chinese Academy of Medical Sciences, Beijing, China (http://anatomy.sbm.pumc.edu.cn/). Brains were dissected and paraffins of human cortex were prepared by trained neuroanatomists with written informed consent. Our research complied with all ethical regulations approved by the Ethics Committee of Shanghai Jiao Tong University School of Medicine. The detailed information of non-demented control and AD patients was represented in Table S2.

### Mice and ethics statement

APP/PS1 transgenic mice (expressing a chimeric mouse/human APP695 Swedish mutation and a human PS1 mutation) and wild-type (WT) C57BL/6J mice were purchased from Changzhou Cavens Model animal Co. Ltd (Changzhou, China) and randomly allocated to different groups. All mice used in experiments were male. Mice were housed under conditions of constant temperature and humidity, with free access to food and water in a 12-h light/dark cycle. All animal experiments were performed in accordance to protocols approved by the Ethics Committee of Shanghai Jiao Tong University School of Medicine. Investigators were blinded to the group allocation.

### Microglia Isolation

After mice were deeply anesthetized, blood was extracted by ventricular puncture and mice were perfused with PBS (#10010023; Thermo Scientific, Waltham, MA, USA). The brain tissue of mice was temporarily placed in ice-cold HBSS (#14175095; Thermo Scientific). Tissue was then dissociated and digested for 15 min at 37 °C by Papain (2 mg/mL, LS003126; Worthington, Lakewood, NJ) in RPMI 1640 medium (#11875093; Gibco, Carlsbad, CA, USA). The mixture passed through a 70 μm filter. Dispersed cells were harvested by centrifugation at 800 rpm for 10 min at 4 °C. The cell pellet was resuspended in a continuous 30% Percoll (#P990025; Macklin, Shanghai, China) gradient at 700 g for 15 min. For microglia isolation, Dynabeads Biotin Binder (#11047; Invitrogen, Carlsbad, CA, USA) was pre-incubated with anti-CD11b antibody (#13-0112-82; Invitrogen) for 30 min at room temperature (RT), and then incubated with cells for 30 min at 4 °C with gentle tilting and rotation. Microglia were then collected by magnetic sorting.

### CircRNA microarray assay

Three samples of cortical microglia from 6-month-old male APP/PS1 mice and three samples of cortical microglia from 6-month-old male WT mice were used for mouse circRNA microarray detection by Aksomics (Shanghai, China). Briefly, total RNA from isolated cortical microglia was extracted by Trizol reagent (#15596018CN; Invitrogen). RNA quantity and quality were assessed at A260/A280 nm by NanoDrop (NanoDrop, Wilmington, DE, USA) and Agilent 2100 (Agilent, Palo Alto, CA, USA). Total RNAs were digested using Rnase R (#RNR07250; Epicentre, Madison, Wisconsin, USA) to eliminate linear RNAs and enrich circRNAs. Enriched circRNAs were amplified and transcribed into fluorescent cDNA using an Arraystar Super RNA Labeling Kit (Arraystar, Rockville, MD, USA) by a random priming method. Then the labeled cRNAs were hybridized onto the Arraystar Mouse circRNA Array V2 (8×15K, Arraystar) and scanned by the Agilent Scanner G2505C. Array images were analyzed by Agilent Feature Extraction software. Quantile normalization and data processing were conducted using the R software limma package.

### Immunostaining

After deeply anesthetized, mice were perfused with 0.9% ice-cold saline (#MA0083; Meilun, Dalian, China), followed by 4% paraformaldehyde (#MA0192; Meilun). Brain samples were collected, immersed in 4% paraformaldehyde overnight, and then transferred to 20% sucrose for three days and 30% sucrose for three days at 4 °C. Brain samples were then prepared for 20 μm frozen sections using the Leica CM1950 Cryostat (Leica, Wetzlar, Germany). For immunofluorescent staining of mice brain, sections were washed using PBS. For cellular immunofluorescent staining, cells were washed using PBS, fixed using 4% paraformaldehyde at RT for 15 min, and washed using PBS. Then brain sections/cells were permeabilized using Triton X-100 (#P0096; Beyotime, Shanghai, China) in PBST (PBS with 0.1% Tween 20) for 15 min at RT. Brain sections/cells were blocked with a solution of 5% BSA (#ST023; Beyotime) in PBS for 1 h at RT, followed with incubation with a primary antibody overnight at 4 °C. Subsequently, brain sections/cells were incubated with a secondary antibody for 1 h at RT. The DAPI fluorescent dye (#62248; Thermo Scientific) was used to stain the nuclei. Fluorescent images were captured by a Leica SP8 confocal microscope (Leica, Wetzlar, Germany). The antibodies used were listed in Table S3.

### Fluorescence in situ hybridization (FISH)

FISH kits for cell climbing tablets, frozen sections of mice brain, and paraffin sections of human cortex were purchased from Genepharma (Shanghai, China). FISH was performed according to manufacturer's instructions. Oligonucleotide-modified probe sequences for circDlg1 and circDLG1 were synthesized by Genepharma (Shanghai, China). The probes were hybridized with brain sections/BV-2 cells/HMC3 cells for 18 h at 37 °C. Fluorescent images were captured by a Leica SP8 confocal microscope (Leica, Wetzlar, Germany). Images were analyzed using Image J (NIH, Bethesda, MD, USA). CircDlg1/circDLG1 countings were marked and calculated using the “Cell Counter” plugin of Image J 17, 85. The number of neuron/microglia/astrocyte was counted according to nuclei (DAPI) completely colocalized with NeuN/Iba1/GFAP staining. CircDlg1/circDLG1 countings^+^ per cell was manually counted based on the colocalization of circDlg1/circDLG1, NeuN/Iba1/GFAP and DAPI. The probe sequences were listed in Table S4.

### Tyramide signal amplification (TSA)

Mice brain frozen sections were firstly permeabilized using Triton X-100 in PBST. Then, sections were heated at medium heat for 8 min, unheated for 8 min, and heated at medium low heat for 7 min in EDTA antigen repair solution (pH 9.0) (#G1203; Servicebio, Wuhan, China). After cooling, sections were blocked with 3% H_2_O_2_ solution for 15 min and 5% BSA solution for 1 h at RT, followed with incubation with a primary antibody overnight at 4 °C. Sections were then incubated with a secondary antibody labeled by HRP for 1 h at RT. Tyramide dye (#AFIHC024; AiFang biological, Changsha, China) was applied to amply target protein signal for 10 min at RT. Sections were then transferred to antibody eluent specific for mIHC (#abs994; absin, Shanghai, China) and heated for 15 min at 37 °C to remove the primary and secondary antibodies that have been incorporated into the tissue. Then the other primary antibody was used and steps were repeated until all target proteins were labeled. The DAPI fluorescent dye was used to stain the nuclei. Fluorescent images were captured by a digital pathology scanner (KFBIO, Yuyao, China). The antibodies used were listed in Table S3.

### Quantitative real-time PCR (qRT-PCR)

Total RNA from cell and mouse tissue was extracted using Trizol reagent. Nuclear and cytoplasmic RNA were extracted using Cytoplasmic & Nuclear RNA Purification kit (#21000; Norgen, Thorold, Canada). RNA was reverse-transcribed into cDNA using PrimeScript^TM^ RT Master Mix (#RR036A; TAKARA, Kyoto, Japan). qRT-PCR was performed using TB Green^TM^ Premix Ex Taq^TM^ (#RR420A; TAKARA) on LightCycler480 System (Roche, Basel, Switzerland). The primers were synthesized by Ribobio (Guangzhou, China) and listed in Table S4.

### Stereotactic injection

After mice were anesthetized, the head was shaved and secured in the stereotaxic injection apparatus (RWD Life Science, Shenzhen, China). Adeno-associated virus9 (AAV9) preparations expressing short hairpin RNA (shRNA) with Enhanced Green Fluorescent Protein (EGFP) signal under the Iba1 promoter (Iba1-shRNA, 1×10^11^ viral genomes for each mouse) were microinjected into the lateral ventricle (from bregma, anteriorposterior: -0.3 mm; lateral: ±1 mm, ventral: -2.2 mm) using a microliter syringe (Hamilton, Bonaduz, Switzerland) in 10 min. The microliter syringe was left in place for 10 min to avoid backflow along the pipette track. AAV9 preparations expressing Iba1-sh-circCon, Iba1-sh-circDlg1, Iba1-shCon, and Iba1-shPDE4B were constructed and packaged by Genomeditech Co. Ltd (Shanghai, China).

### Microglial morphology and spatial analysis

Microglial images were captured at 1 μm intervals and each maximum intensity projection image was acquired by processing consecutive Z-stack images using a Leica SP8 confocal microscope (Leica, Wetzlar, Germany). Images were denoised to optimize cellular segmentation. For each microglia, concentric circles were drawn at the center of the soma with a 0.5 μm step. Then Sholl analysis was performed to create a Sholl plot using Image J. Ramifications per cell and ramification length were determined as previously reported 86. Briefly, images were converted into representative binary and skeletonized images for morphology data using a AnalyzeSkeleton (2D/3D) plugin of Image J. For each Aβ plaque, the total number of microglia and Iba1 coverage within a circular area of 30 μm centered on an Aβ plaque were quantified using Image J.

### Behavior tests

Mice were placed in the testing room 2 h before behavior tests to acclimate. All behavioral tests were carried out between 9:00 and 17:00 in a quiet room with dim light and recorded by a video camera (BASLER, Ahrensburg, Germany). Collected data were analyzed by EthoVision XT16 software (Noldus, Wageningen, Netherlands). For spontaneous alternation analysis in the YM task, an opaque perspex YM device (20 cm in length, 15 cm in width, and 15 cm in height) comprising three identical arms with an angle of 120° was used. Each mouse explored freely for 5 min from the end of the same arm. Total arm entries and the spontaneous alternation were recorded and analyzed. The spontaneous alternation was defined as the number of consecutive entries into three different arms divided by the number of possible alternations.

For novel recognition index analysis in the NOR task, an NOR arena (60 cm in length, width, and height) containing two objects was applied. Mice were placed in the arena without objects for 5 min to acclimate. On the training day, mice were allowed to explore two same objects for 3 min. On the testing day, one familiar object was maintained and the other familiar object was replaced by a new object. Mice explored two different objects for 3 min. The time that mice spent on exploring different objects were recorded and the recognition index was analyzed. Recognition index was defined as the time that mice spent on exploring the new object divided by the total exploratory time.

For the MWM task, a black circular tank (diameter of 120 cm, 50 cm in height, and 25 cm in depth) filled with opaque water (22 ± 1 °C) was used. A hidden platform was submerged 1.5 cm underwater. During the 4/5-day training phase, mice performed four training trials from 4 quadrants to learn to find the hidden platform within 60 s per day. Each mouse could stay on the platform for 10 s if the platform was found. Each mouse was guided to the platform and stayed there for 10 s if the mouse failed to find platform within 60 s. The probe trial was performed 24 h after the last training trail without the platform. Each mouse was subjected to the quadrant that was opposite the platform. The performance recorded within 60 s was used to evaluate learning and spatial memory.

### Cell cultures

Human embryonic kidney HEK293 cells, and BV-2 cells were cultured in DMEM (#11965092; Gibco) which was supplemented with 10% FBS (#10099141C; Gibco) and 1% penicillin-streptomycin mix (#15140122; Gibco). Primary microglia were isolated as previously reported 87. Briefly, cortices without meninges from C57BL/6 mice aged P0-P3 was homogenized in DMEM, filtered through a 70 μm filter, and cultured in DMEM supplemented with 10% FBS and 1% penicillin-streptomycin mix on poly-L-lysine-coated flasks. After a 24 h incubation, the medium was changed. The primary microglia were harvested by shaking (200 rpm, 4 h) 10-14 days after culture and subjected to various experiments within 24 h. All cells were maintained at 37 °C in a humidified 5% CO_2_ atmosphere. Cells were seeded into 6-well/12-well/24-well plates for experiments.

### Aβ_42_ phagocytosis assay *in vitro*

Human FAM-labeled Aβ_42_ (Aβ_42_-FAM) was obtained from Anaspec (#AS-23525-05; Anaspec, Fremont, CA, USA), reconstituted as the manufacturer's instruction with 1.0% ammonium hydroxide (#AS-61322; Anaspec), followed by dilution in PBS to 1 mg/ml and aggregation at 4 °C for 24 h. For phagocytosis assay *in vitro*, BV-2 cells and primary microglia were treated with siRNAs for 24 h, followed by 10 µM Aβ_42_-FAM stimulation for 24 h. Aβ_42_-FAM uptake was detected by the immunostaining protocol. For Aβ_42_-FAM uptake detected by flow cytometry, single-cell suspensions were prepared in PBS supplemented with 2% FBS and 0.5% BSA. Cells without Aβ_42_-FAM treatment were used as compensation controls to avoid any non-specific signals. Data were acquired on a Attune NxT Acoustic Focusing Cytometer (Thermo Scientific) and analysed using Flowjo (Version 10; TreeStar, Ashland, OR, USA).

### Western blotting (WB)

Protein lysates from cells and mouse tissue were extracted using RIPA buffer (#P0013B; Beyotime) with Protease Inhibitor Cocktail (#GRF101, Epizyme, Shanghai, China) at 4 °C for 30 min. Supernatants were collected by centrifugation at 16,000 g for 10 min at 4 °C. Protein concentrations were measured using the Pierce^TM^ BCA Protein Assay Kits (#23227; Thermo Scientific). Supernatants containing proteins were then subjected to SDS-PAGE and transferred to polyvinylidene fluoride membranes (#IPVH00010; Millipore, Billerica, MA, USA). Membranes were blocked with 5% BSA-TBST at RT for 1 h, followed by incubation with primary antibody overnight at 4 °C. Membranes were then incubated with a goat anti-rabbit or anti-mouse IgG HRP-conjugated secondary antibody (#A0208/A0216; Beyotime). Odyssey Image Station (LI-COR, Lincoln, Nebraska, USA) detected the protein signal. The antibodies used were listed in Table S3.

### Co-immunoprecipitation (Co-IP)

The ubiquitination of PDE4B and the interactions between PDE4B and Smurf2 were confirmed by Co-IP. Cell lysates were extracted using weak RIPA lysis buffer (#P0013D; Beyotime). The supernatants were collected by centrifugation at 12,000 g for 30 min at 4 °C and incubated with Protein A/G agarose (#20422; Thermo Scientific) for 1 h at 4 °C. The supernatants were collected by magnetic separation, followed by incubation with 3.5 μg antibody overnight at 4 °C. Protein A/G agarose was added to pull down the immune complexes for 1 h on a shaker at 4 °C. Whole-cell extracts and immunoprecipitates were collected for WB analysis. The antibodies used were listed in Table S3.

### siRNA and plasmid transfection

CircDlg1, Flag-PDE4B1, Flag-PDE4B2, Flag-PDE4B3, and truncations of Flag-PDE4B1 plasmids were purchased from Genomeditech Co. Ltd (Shanghai, China). siRNAs targeting circDlg1 and PDE4B were purchased from Genepharma (Shanghai, China). When the confluence of cells reached 70%-80%, cells were transfected with 2.5 μg/mL plasmids or 100 nM siRNAs in Opti-MEM™ (#31985070; Gibco) using Lipofectamine 3000 (#L3000150; Invitrogen). The transfected cells were collected at 24 h for RNA extraction and at 48 h for protein analysis. The sequences of siRNAs were listed in Table S4.

### RNA pull down and mass spectrometry

The Biotin-labeled circDlg1 probes were synthesized by SunBio Biomedical Technology Co., Ltd (Shanghai, China) and listed in Table S3. Cell lysates and cortical tissue of WT mice were extracted using weak RIPA lysis buffer with a mixture of Protease Inhibitor Cocktail and Rnase inhibitor (#R0101; Beyotime) at 4 °C for 30 min. Supernatants were collected by centrifugation at 12,000 g for 30 min at 4 °C, followed by incubation with 4.5 μg biotinylated probes at RT for 1 h. Lysates were then incubated with streptavidin magnetic beads (#88817; Invitrogen) at RT for 1 h. The RNA-protein complex was pulled down by magnetic separation and analyzed by WB or MS analysis conducted by SunBio Biomedical Technology Co., Ltd (Shanghai, China).

### RNA immunoprecipitation (RIP)

RIP experiments were conducted using a Magna RIP^TM^ RNA-Binding Protein Immunoprecipitation Kit (#17-704; Millipore). Cell lysates were extracted using weak RIPA lysis buffer with a mixture of Protease Inhibitor Cocktail and Rnase inhibitor at 4 °C for 30 min, followed by treatment with magnetic beads coated with 5 μg of specific antibodies against mouse IgG or PDE4B overnight at 4 °C. The immunoprecipitated RNAs were further detected by qRT-PCR. The antibodies used were listed in Table S2 and the primers were shown in Table S4.

### ELISA

Cell lysates were extracted by 3 times of rapid freeze-thawing by ice-cold PBS. Supernatants were collected by centrifugation at 3000 rpm for 20 min at 4 °C. The BCA method was applied to measure protein concentrations using the Pierce^TM^ BCA Protein Assay Kits. cAMP was measured by a commercially available ELISA kit (AB-W30665; Abmart, Shanghai, China) according to the manufacturer's instructions. Absorbance was detected at 450 nm on a Varioskan Flash (Thermo Scientific).

### Drug treatment

BV-2 cells were treated with LPS (#L2880; Sigma, St. Louis, MO, USA) at a concentration of 100 ng/ml for 18 h for qRT-PCR or WB. BV-2 cells were treated with AcD (#HY-17559; MedChemExpress, Shanghai, China) at a concentration of 2 μg/ml for indicated time points (0, 4, 8, and 12 h or 0, 30, 60, 90, 120, 180 min) for qRT-PCR. RNA from BV-2 cells was extracted and incubated with 3 U/μg of RNase R (#R7092; Beyotime) for 10 min at 37 °C to detect circDlg1 and Dlg1 level. BV-2 cells were transfected with si-NC or si-circDlg1 followed by treatment of CHX at a concentration of 10 μg/ml for indicated time points (0, 30, 60, 120 and 180 min) for WB. BV-2 cells were transfected with si-NC or si-circDlg1 followed by the treatment of MG-132 (10 μM, HY-13259; MedChemExpress)/Bort (200 nM, #HY-10227; MedChemExpress)/Chlo (10 μM, #HY-17589A; MedChemExpress) for 1 h for WB.

### Statistical analysis

All data were presented by at least three biologically independent experiments. All results were analyzed using GraphPad Prism 8.0 and shown as mean ± SEM. Statistical tests included two-sided unpaired Student's t test for two groups and two-way analysis of variance (ANOVA) followed by Tukey's post hoc test for multiple comparisons. Correlation was calculated using Pearson correlation coefficients. Linear regression analysis was applied to assess the correlation between factors. Results were statistically significant when *P* < 0.05.

## Supplementary Material

Supplementary figures and tables 2-4.

Supplementary table 1.

## Abbreviations

AAV9: adeno-associated virus9
AcD: actinomycin D
AD: Alzheimer's disease
AUC: area under the curve
Aβ: amyloid β
Bort: bortezomib
Chlo: chloroquine
CHX: cycloheximide
circRNAs: circular RNAs
CNS: central nervous system
Co-IP: co-immunoprecipitation
coloc.: colocalization
Con: control
DAM: disease-associated microglia
EGFP: enhanced green fluorescent protein
FISH: fluorescence in situ hybridization
FSC: forward and side scatter
i.c.v.: intracerebroventricular
iBAQ: intensity based absolute quantification
LPS: lipopolysaccharide
MACS: magnetic-activated cell sorting
miRNAs: microRNAs
MS: mass spectrometry
MWM: Morris water maze
NC: negative control
NOR: novel object recognition
PDE: phosphodiesterase
qRT-PCR: quantitative real-time PCR
RT: room temperature
RIP: RNA immunoprecipitation
SSC-A: side scatter area
SSC-H: side scatter height
Ub: ubiquitin
WB: western blot
WT: wild-type
YM: Y-maze
