# Supplementary material for: Microglial circDlg1 modulates neuroinflammation by blocking PDE4B ubiquitination-dependent degradation associated with Alzheimer's disease
Source: Theranostics. 2025 Feb 24;15(8):3401–23. doi: 10.7150/thno.104709 (PMC11905123; doi:10.7150/thno.104709)
Supplement: Supplementary file 1 — Supplementary figures and tables 2-4. [file thnov15p3401s1.pdf]

Supplementary information

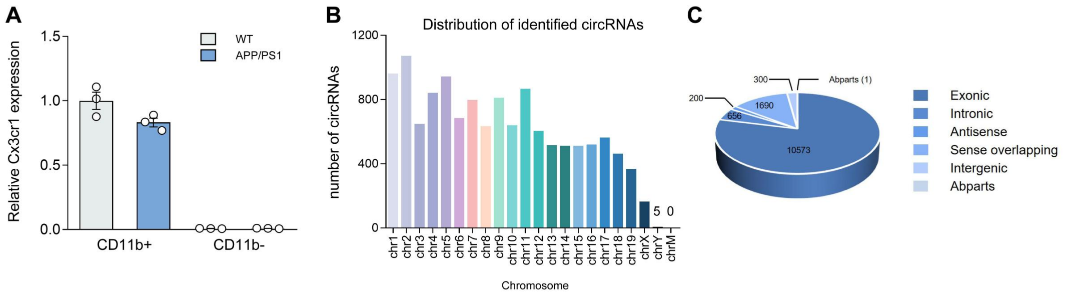

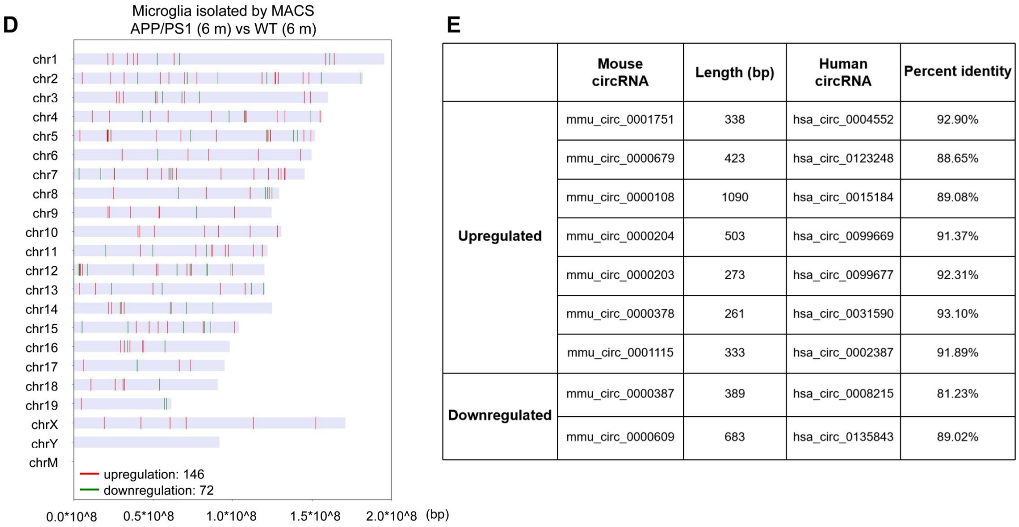

E

	Mouse circRNA	Length (bp)	Human circRNA	Percent identity
Upregulated	mmu_circ_0001751	338	hsa_circ_0004552	92.90%
	mmu_circ_0000679	423	hsa_circ_0123248	88.65%
	mmu_circ_0000108	1090	hsa_circ_0015184	89.08%
	mmu_circ_0000204	503	hsa_circ_0099669	91.37%
	mmu_circ_0000203	273	hsa_circ_0099677	92.31%
	mmu_circ_0000378	261	hsa_circ_0031590	93.10%
	mmu_circ_0001115	333	hsa_circ_0002387	91.89%
Downregulated	mmu_circ_0000387	389	hsa_circ_0008215	81.23%
	mmu_circ_0000609	683	hsa_circ_0135843	89.02%

Figure S1. CircRNA microarray is applied to detect the expression of circRNA, followed by circBase identification, conservative analysis, and length screening. (A) qRT-PCR assays for the relative expression of Cx3cr1 in CD11b^{+} and CD11b^{-} cells isolated from the cortex of 6-month-old male WT and APP/PS1 mice to validate microglia isolation (n = 3 mice per group). (B) Distribution of the identified circRNAs of cortical microglia on mouse chromosomes. X-axis: name of chromosomes; Y-axis: the number of circRNAs. (C) Composition of the identified circRNAs in terms of genomic origin. (D) Distribution of the identified differentially expressed circRNAs (fold change $>$ 1.5, $P <$ 0.05) on mouse chromosomes (n = 3 mice per group). X-axis: the length of DNA; Y-axis: name of chromosomes; downregulation: green lines; upregulation: red lines. (E) The differentially expressed circRNAs, which were recorded in circBase and within 200-2000 bp in length, were conservatively analyzed. Data were presented as mean ± SEM.

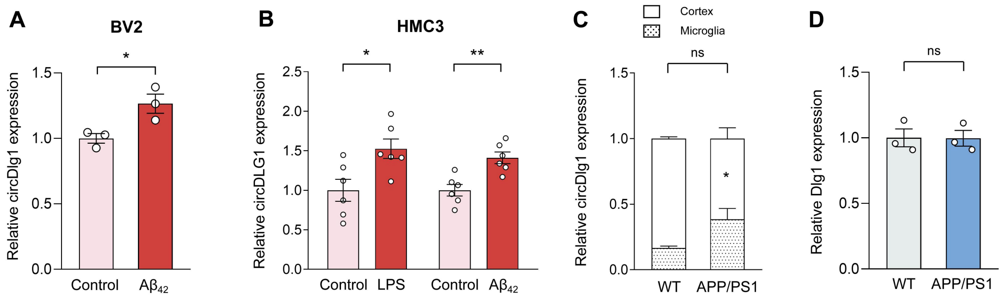

Figure S2. Expression of circDlg1 and Dlg1. (A) qRT-PCR assays for the relative expression of circDlg1 in BV-2 cells treated with $A\beta_{42}$ (10 μM) for 24 h (n = 3 biologically independent experiments). (B) qRT-PCR assays for the relative expression of circDLG1 in HMC3 cells treated with $A\beta_{42}$ (10 μM) for 24 h or LPS (100 ng/ml) for 18 h (n = 6 biologically independent experiments). (C) qRT-PCR assays for the relative expression of circDlg1 in the cortex of 6-month-old male WT and APP/PS1 mice (n = 3 mice per group). CircDlg1 expression in microglia accounted for circDlg1 expression in cortex was shown (n = 3 mice per group). (D) qRT-PCR assays for the relative expression of Dlg1 in cortical microglia isolated from 6-month-old male WT and APP/PS1 mice (n = 3 mice per group). Data were presented as mean ± SEM. Two-tailed t-tests were used. $^{*}P < 0.05$, $^{**}P < 0.01$.

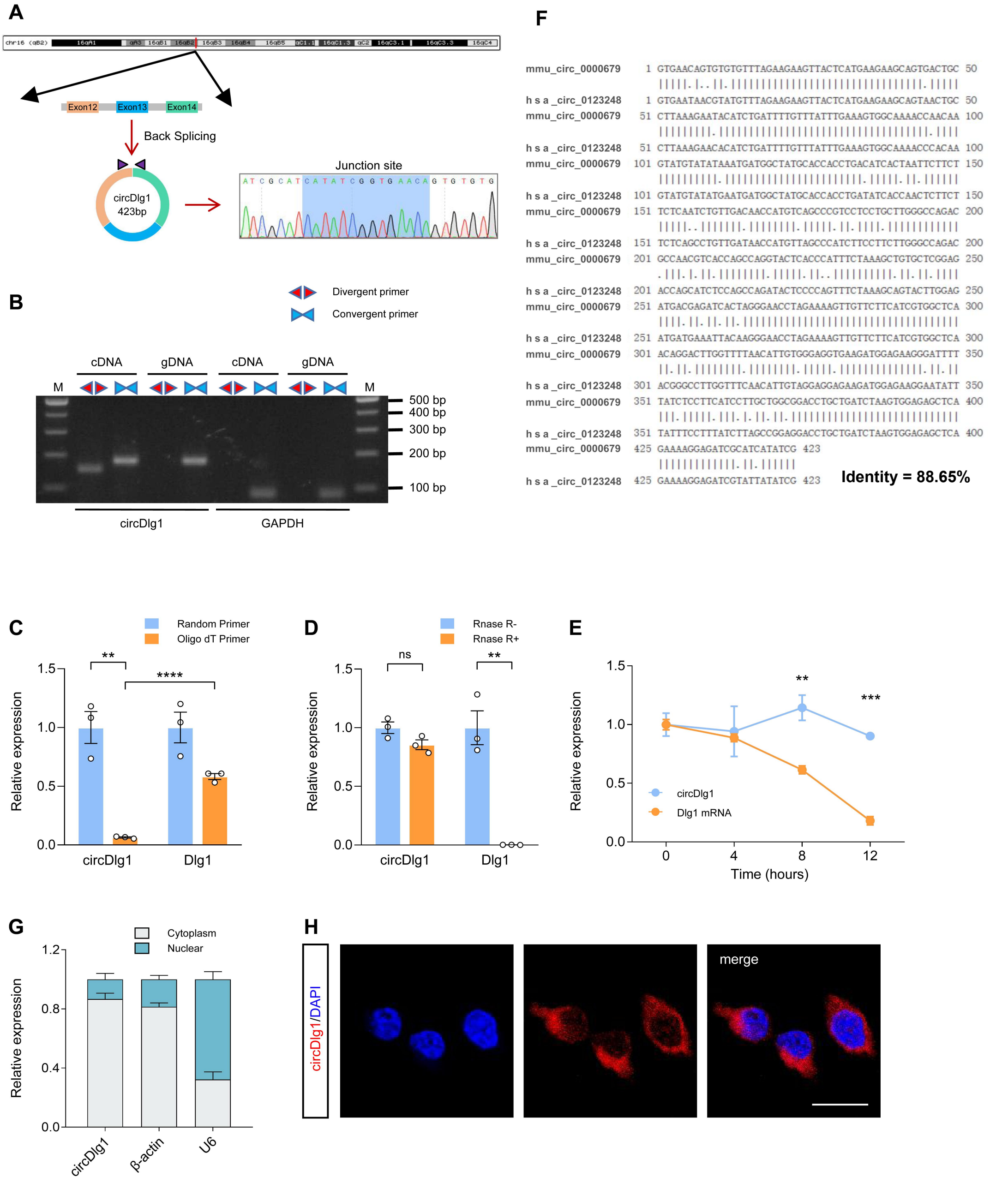

Figure S3. Identification of circDlg1. (A) The schematic illustration showed the circularization of circDlg1 from exons 12, 13 and 14 of Dlg1 gene by back splicing. The back-splicing junction of circDlg1 was verified by Sanger sequencing. (B) Convergent or divergent primers were used to detect circDlg1 in BV-2 cells by agarose gel electrophoresis. CircDlg1 could be amplified by divergent primers in cDNA but not genomic DNA (gDNA). GAPDH was used as linear control. M: marker. (C) qRT-PCR assays for the relative expression of circDlg1 and linear Dlg1 using the template cDNA reverse-transcribed from RNA of BV-2 cells by random primers and oligo dT primers (n = 3 biologically independent experiments). (D) qRT-PCR assays for the relative expression of circDlg1 and

linear Dlg1 in BV-2 cells treated with RNase R (n = 3 biologically independent experiments). (E) qRT-PCR assays for the relative expression of circDlg1 and linear Dlg1 in BV-2 cells treated with AcD (2 μg/mL) at the indicated time points (n = 3 biologically independent experiments). AcD: Actinomycin D. Statistical analysis was performed by two-way ANOVA followed by Tukey's post hoc test. $^{**}P < 0.01$, $^{***}P < 0.001$ versus Dlg1 group. (F) Pairwise alignment of the human and mouse circDlg1 sequences. (G) qRT-PCR assays for the relative expression of circDlg1 in cytoplasm and nucleus of BV-2 cells (n = 3 biologically independent experiments). β-actin was used as a positive control of RNA distributed in the cytoplasm. U6 was used as a positive control of RNA distributed in the nucleus. (H) Localization of circDlg1 in BV-2 cells was detected by FISH (n = 3 biologically independent experiments). Scale bar = 20 μm. Data were presented as mean ± SEM. Two-tailed t-tests were used unless otherwise specified. $^{**}P < 0.01$, $^{****}P < 0.0001$.

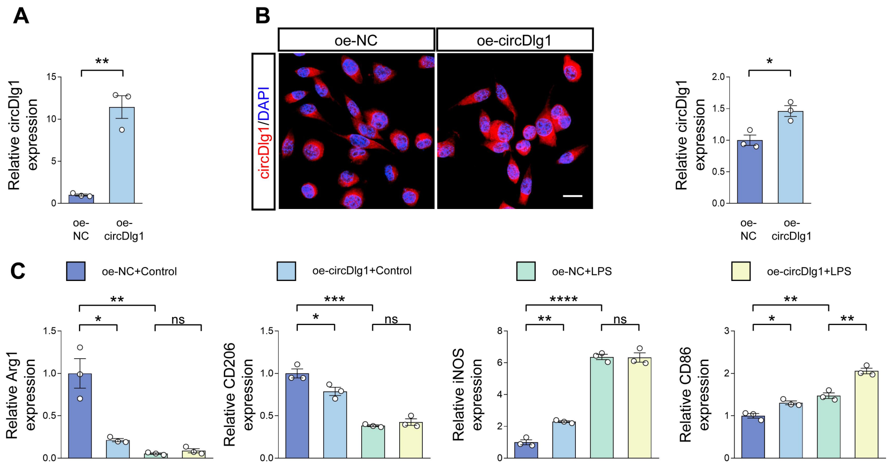

Figure S4. Overexpression of circDlg1 facilitates microglial M1 polarization *in vitro*. (A) qRT-PCR assays for the relative expression of circDlg1 in BV-2 cells transfected with oe-NC or oe-circDlg1 (n = 3 biologically independent experiments). (B) The expression of circDlg1 in BV-2 cells was detected by FISH. Relative fluorescence intensity of circDlg1 was quantified on the right (n = 3 biologically independent experiments). Scale bar = 20 μm. (C) qRT-PCR assays for the relative expression of Arg1, CD206, iNOS, and CD86 in BV-2 cells transfected with oe-NC or oe-circDlg1 followed by treatment of LPS (100 ng/ml) for 18 h (n = 3 biologically independent experiments). Data were presented as mean ± SEM. Two-tailed t-tests were used. $^{*}P < 0.05$, $^{**}P < 0.01$, $^{***}P < 0.001$, $^{****}P <$

0.0001.

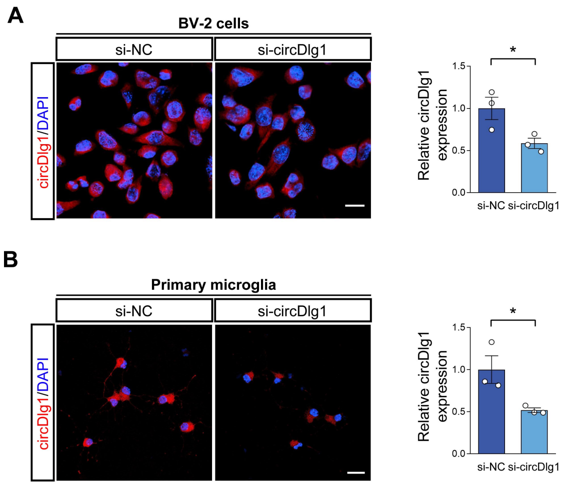

Figure S5. Expression of circDlg1. (A) The expression of circDlg1 in BV-2 cells transfected with si-NC or si-circDlg1 was detected by FISH. Relative fluorescence intensity of circDlg1 was quantified on the right (n = 3 biologically independent experiments). Scale bar = 20 μm. (B) The expression of circDlg1 in primary microglia transfected with si-NC or si-circDlg1 was detected by FISH. Relative fluorescence intensity of circDlg1 was quantified on the right (n = 3 biologically independent experiments). Each dot of primary microglia represented cells pooled from 6-8 neonatal brains. Scale bar = 20 μm. Data were presented as mean ± SEM. Two-tailed t-tests were used. *$P < 0.05$.

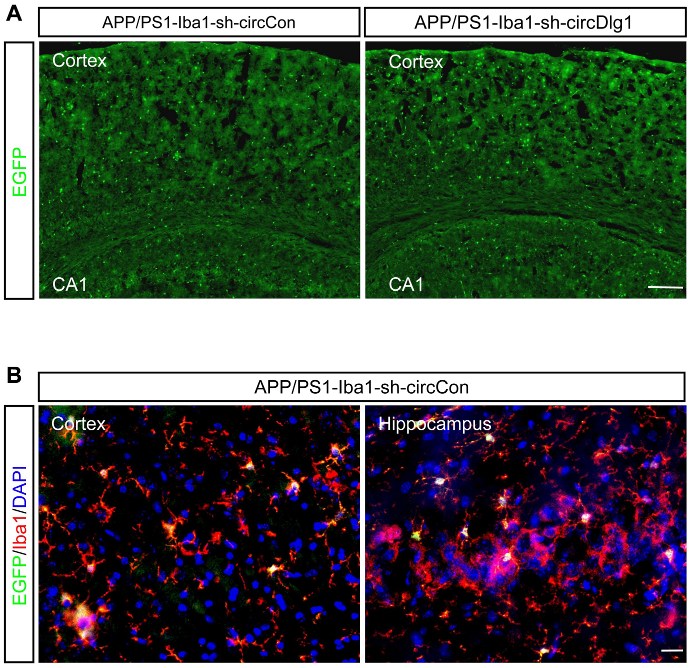

Figure S6. Visualization of the diffusion of AAV9 preparations in the brain of APP/PS1 mice. (A) Immunostaining for EGFP was performed to visualize AAV9 viral diffusion in APP/PS1 mice injected with AAV9-Iba1-sh-circCon or AAV9-Iba1-sh-circDlg1 (n = 3 mice per group). Scale bar = 200 µm. (B) Immunostaining was performed to detect the colocalization between EGFP and microglia in the cortex and hippocampus of APP/PS1 mice injected with AAV9-Iba1-sh-circCon (n = 3 mice per group). Scale bar = 20 µm.

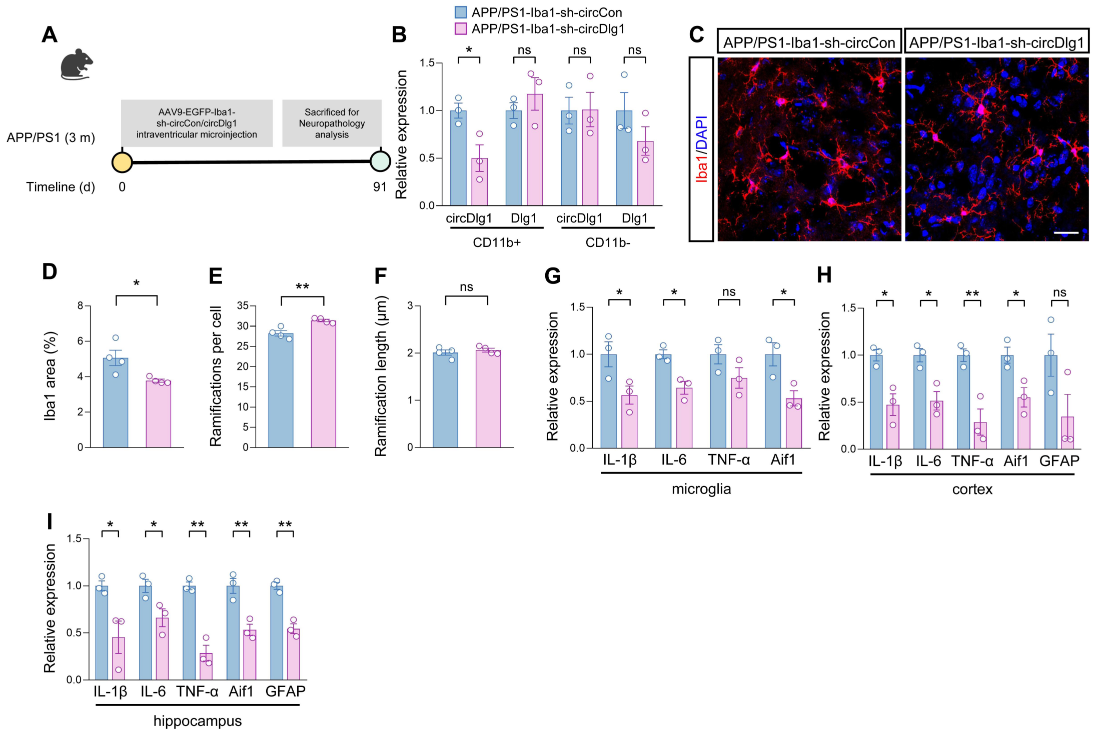

Figure S7. Microglia-specific knockdown of circDlg1 prevents microglial dysfunction and neuroinflammation in the early pathological stage of APP/PS1 mice. (A) Experimental schematic of 3-month-old male APP/PS1 mice. (B) qRT-PCR assays for the relative expression of circDlg1 and Dlg1 in CD11b$^+$ and CD11b$^-$ cells isolated from the brains of APP/PS1 mice injected with AAV9-Iba1-sh-circCon or AAV9-Iba1-sh-circDlg1 (n = 3 mice per group). (C) Representative images of microglia in the cortex of APP/PS1 mice injected with AAV9-Iba1-sh-circCon or AAV9-Iba1-sh-circDlg1. Scale bar = 20 μm. (D-F) Total Iba1 area in the cortex (D) and skeletal analysis of microglia including ramifications per cell (E) and each ramification length (F) in (C) were quantified (n = 4 mice per group). (G-I) qRT-PCR assays for the relative expression of neuroinflammation-related genes in microglia (G), cortex (H), and hippocampus (I) of APP/PS1 mice injected with AAV9-Iba1-sh-circCon or AAV9-Iba1-sh-circDlg1 (n = 3 mice per group). Data were presented as mean ± SEM. Two-tailed t-tests were used. *$P < 0.05$, **$P < 0.01$.

A

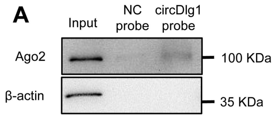

B

Sequence Name	RNA Size (bp)	ORF Size (bp)	Ficket Score	Hexamer Score	Codiing Probability	Coding Label
mmu_circ_0000679	423	321	1.07	0.09	0.35	no

C

Gene	Mw (kDa)	Length	iBAQ (%)	MS/MS count
Ap2a2	103.960	939	0.40	1
Hnrnpu	90.584	825	0.75	4
PDE4B	83.343	736	34.44	12
Cep83	82.940	701	0.60	0
Jup	81.745	745	0.29	1
Caprin1	78.366	709	1.23	2
Sfpq	76.149	707	18.12	12
Nono	54.232	471	0.31	2
Hnrnpk	50.976	463	0.34	1
Tubb2a	49.907	445	5.73	2
Tuba1c	49.895	449	0.54	2
Ap2m1	49.655	435	0.51	2
Tubb4a	49.586	444	0.82	1
Csnk1e	47.315	416	0.44	1
Hnrnpg	42.332	391	2.67	7
Glul	42.064	373	0.38	1
Actg1	41.793	375	0.54	1
Kcnab2	41.000	367	1.01	4
Hnrnpa3	39.595	378	7.16	3
Hnrnpa1	38.747	372	11.56	3
Hnrnpa2b1	37.430	353	3.04	6
Mdh2	35.503	338	0.56	1
Pura	34.911	322	6.26	5
Arg1	34.735	322	0.54	1
Hnrnpc	33.670	306	0.66	1

D

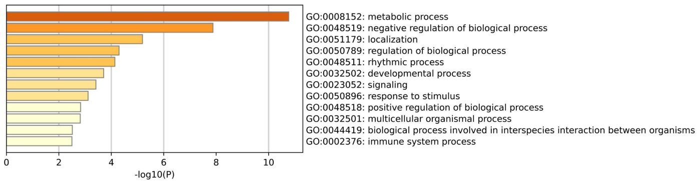

E

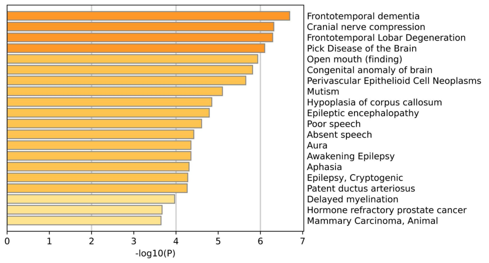

Figure S8. Analysis of proteins interacting with circDlg1 detected by RNA pulldown assays combined with MS and WB. (A) WB after RNA pulldown assays using NC or circDlg1 probe was performed to verify the interaction between circDlg1 and Ago2 in the cortex of 6-month-old male WT mice (n = 3 mice). (B) Coding Potential Assessment Tool (http://lilab.research.bcm.edu/calculator_sub.php) was used to analyze the coding potential of circDlg1. (C) The 24 proteins in Figure 5C that interacted with circDlg1 in microglia were listed. (D) GO functional categories of the 24 proteins interacting with circDlg1. (E) Metascape database (metascape.org) performed disease network analysis for the 24 proteins.

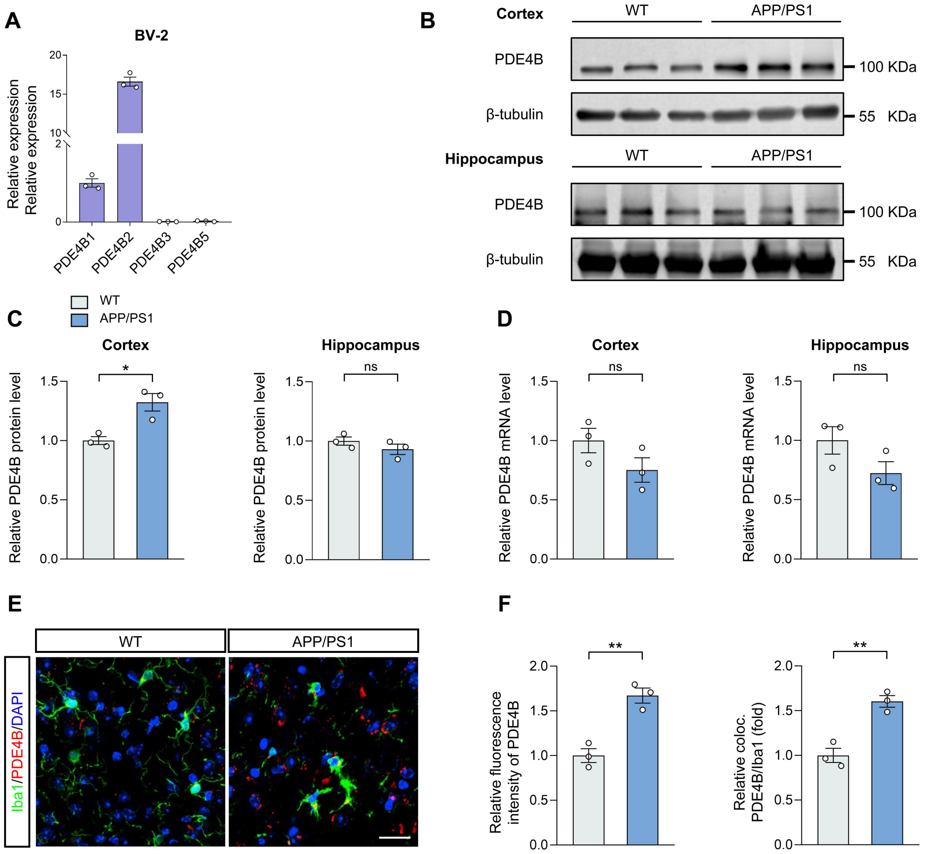

Figure S9. The expression of PDE4B in 6-month-old male WT and APP/PS1 mice. (A) qRT-PCR assays for the relative abundance of PDE4B variants (PDE4B1, PDE4B2, PDE4B3, and PDE4B5) in BV-2 cells (n = 3 biologically independent experiments). (B) Protein expression of PDE4B in the cortex and hippocampus of 6-month-old male WT and APP/PS1 mice was detected by WB (n = 3 mice per group). (C) Relative PDE4B protein levels of (B) were quantified (n = 3 mice per group). (D) qRT-PCR assays for the relative expression of PDE4B in the cortex and hippocampus of 6-month-old male WT and APP/PS1 mice (n = 3 mice per group). (E) Representative cortical images of PDE4B and microglia (Iba1) in brain sections of 6-month-old male WT and APP/PS1 mice. Scale bar = 20 μm. (F) Relative fluorescence intensity of PDE4B in cortex and the relative fold change of PDE4B and microglia coloc. in (E) were quantified (n = 3 mice per group). Data were presented as mean ± SEM. Two-tailed t-tests were used. $*P < 0.05$, $**P < 0.01$.

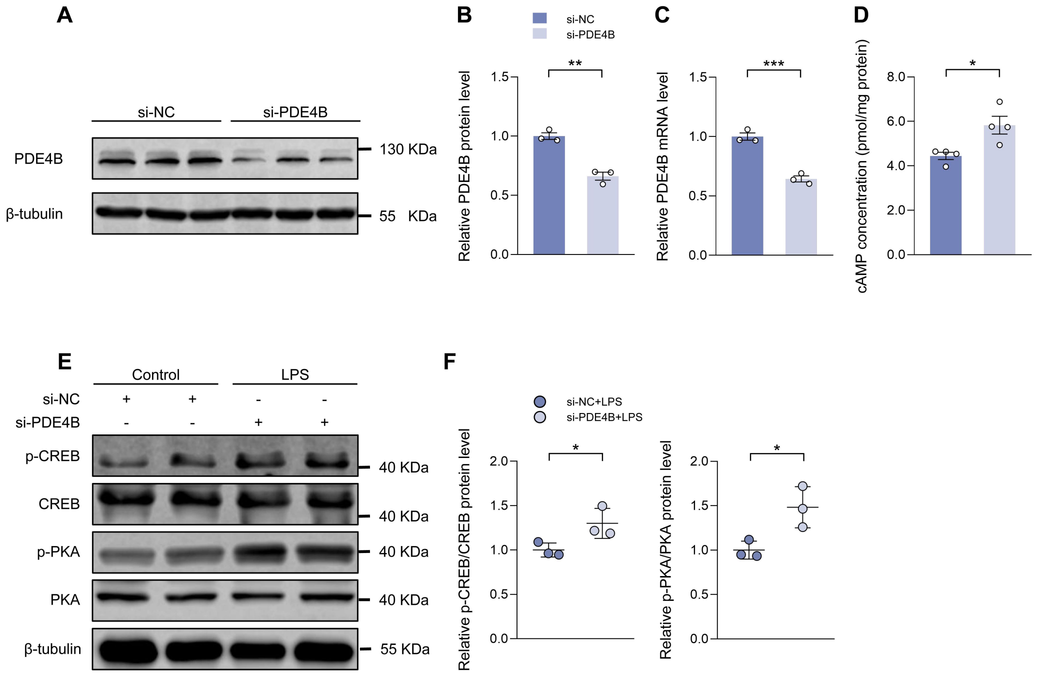

Figure S10. Knockdown of PDE4B activates the cAMP/PKA/CREB signaling pathway in BV-2 cells. (A) Protein expression of PDE4B in BV-2 cells transfected with si-NC or si-PDE4B was detected by WB (n = 3 biologically independent experiments). (B) Relative PDE4B protein levels in (A) were quantified (n = 3 biologically independent experiments). (C) qRT-PCR assays for the relative expression of PDE4B in BV-2 cells transfected with si-NC or si-PDE4B (n = 3 biologically independent experiments). (D) ELISA detected cAMP concentration in BV-2 cells transfected with si-NC or si-PDE4B (n = 4 biologically independent experiments). (E) Protein expression of p-CREB, CREB, p-PKA, and PKA in BV-2 cells transfected with si-NC or si-circDlg1 followed by treatment of LPS (100 ng/ml) for 18 h was detected by WB (n = 3 biologically independent experiments). (F) Relative p-CREB/CREB, and p-PKA/PKA protein levels in (E) were quantified (n = 3 biologically independent experiments). Data were presented as mean ± SEM. Two-tailed t-tests were used. *$P < 0.05$, **$P < 0.01$, ***$P < 0.001$.

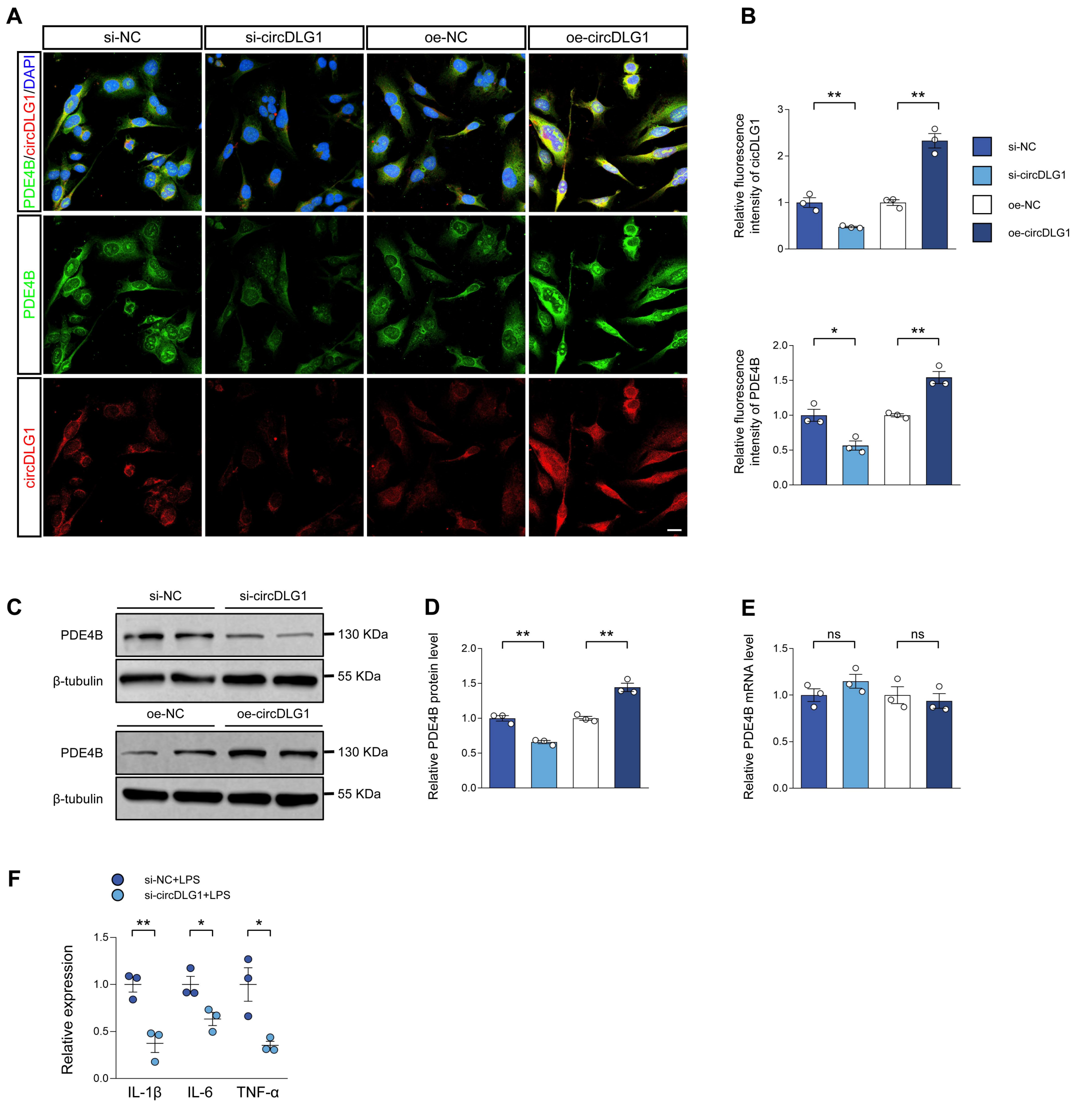

Figure S11. CircDLG1 regulates the PDE4B expression at the protein level but not the RNA level in HMC3 cells. (A) Representative images of PDE4B in HMC3 cells transfected with si-NC, si-circDLG1, oe-NC, and oe-circDLG1. Scale bar = 20 µm. (B) Relative fluorescence intensity of circDLG1 and PDE4B in (A) was quantified (n = 3 biologically independent experiments). (C) Protein expression of PDE4B in HMC3 cells transfected with si-NC, si-circDLG1, oe-NC, and oe-circDLG1 was detected by WB (n = 3 biologically independent experiments). (D) Relative PDE4B protein levels in (C) were quantified (n = 3 biologically independent experiments). (E) qRT-PCR assays for the relative expression of PDE4B in HMC3 cells transfected with si-NC, si-circDLG1, oe-NC, and oe-circDLG1 (n = 3 biologically independent experiments). (F) qRT-PCR assays for the relative expression of IL-1β, IL-6, and TNF-α in HMC3 cells transfected with si-NC or si-circDLG1 followed

by treatment of LPS (100 ng/ml) for 18 h (n = 3 biologically independent experiments). Data were presented as mean ± SEM. Two-tailed t-tests were used. *$P < 0.05$, **$P < 0.01$.

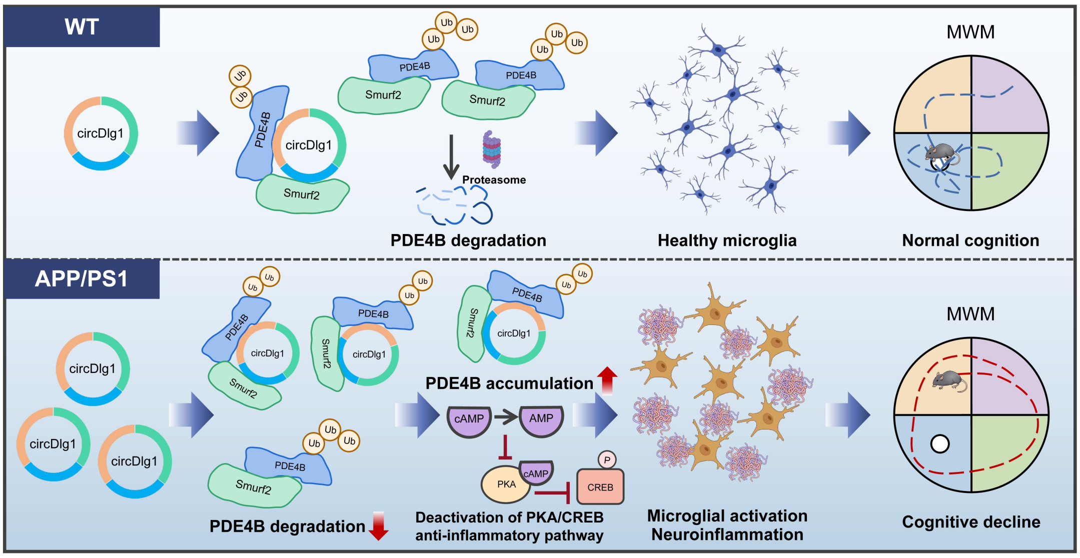

Figure S12. A schematic diagram showing the proposed working model of microglial circDlg1 in APP/PS1 mice. AD pathology triggers upregulation of circDlg1 in microglia, resulting in weakened interaction between PDE4B and Smurf2, an E3 ubiquitin ligase that mediates ubiquitination-dependent degradation of PDE4B. Accumulation of PDE4B leads to degradation of cAMP, deactivation of PKA, microglia dysfunction, neuroinflammation, and cognitive decline and thus promotes AD-associated pathology.

Table S2. Characteristics of cases used for study.

No.	Source	Age (years)	Sex	Diagnosis	Post-mortem delay (h)
1	NHBB	76	Male	Non-demented control	6
2	NHBB	79	Male	Non-demented control	3.5
3	NHBB	67	Male	Non-demented control	5
4	NHBB	80	Male	AD	4.5
5	NHBB	80	Female	AD	18
6	NHBB	85	Female	AD	4.5

NHBB: National Human Brain Bank for Development and Function

Table S3. Antibodies used for study.

Antibodies for WB	Source	Identifier	Dilution
Anti-Argonaute-2	Abcam	ab156870	1:1000
Anti-beta Actin	Abcam	ab227387	1:1000
Anti-beta Tubulin	Abcam	ab6046	1:1000
CREB	Abcam	ab32515	1:1000
Anti-CREB (phospho S133)	Abcam	ab32096	1:1000
PKA	Proteintech	55382-1-AP	1:1000
Anti-PKA alpha/beta/gamma (catalytic subunit) (phospho T197)	Abcam	ab75991	1:1000
Anti-PURA	Abcam	ab79936	1:1000
Anti-SFPQ	Abcam	ab11825	1:1000
Anti-hnRNPA1	Cell Signaling Technology	8443S	1:1000
Anti-hnRNPG	Cell Signaling Technology	14794S	1:1000
Anti-Smurf2	Cell Signaling Technology	12024	1:1000
Anti-Ubiquitin	Cell Signaling Technology	3936T	1:1000
Anti-DYKDDDDK Tag	Thermo Fisher Scientific	MA1-91878	1:1000
Anti-PDE4B	Thermo Fisher Scientific	40-1400	1:1000
HRP-labeled Goat Anti-Mouse IgG H&L	Beyotime	A0216	1:1000
HRP-labeled Goat Anti-Rabbit IgG H&L	Beyotime	A0208	1:1000

Antibodies for Immunostaining	Source	Identifier	Dilution
Anti-APP/β-Amyloid (NAB228)	Cell Signaling Technology	2450S	1:200
Anti-GFAP	Cell Signaling Technology	3670S	1:200
Anti-Iba1	Abcam	ab178847	1:200
Anti-Lamp1	Abcam	ab24170	1:200
Anti-NeuN	Abcam	ab104224	1:200
Anti-PDE4B for cells	Thermo Fisher Scientific	40-1400	1:200
Anti-PDE4B for brain sections	Thermo Fisher Scientific	MA5-25677	1:150
Goat Anti-Mouse IgG H&L (Alexa Fluor® 488) preadsorbed	Abcam	ab150117	1:1000
Goat Anti-Mouse IgG H&L Alexa Fluor® 555) preadsorbed	Abcam	ab150118	1:1000
Goat Anti-Mouse IgG H&L (Alexa Fluor® 647) preadsorbed	Abcam	ab150119	1:1000
Goat Anti-Rabbit IgG H&L (Alexa Fluor® 488) preadsorbed	Abcam	ab150081	1:1000
Goat Anti-Rabbit IgG H&L (Alexa Fluor® 555) preadsorbed	Abcam	ab150082	1:1000
Goat Anti-Rabbit IgG H&L (Alexa Fluor® 647) preadsorbed	Abcam	ab150083	1:1000

Antibodies for TSA	Source	Identifier	Dilution
Anti-CREB (phospho S133)	Abcam	ab32096	1:3000
Anti-PKA alpha/beta/gamma (catalytic subunit) (phospho T197)	Abcam	ab75991	1:3000
Anti-Iba1	Abcam	ab178847	1:10000

**Antibodies for CO-IP and RIP**	**Source**	**Identifier**
Anti-PDE4B	Thermo Fisher Scientific	40-1400
Rabbit IgG	Abmart	B30011M

Table S4. Sequences of FISH probes, RNA pull down probes, siRNAs, and qPCR primers used for study.

FISH probes	Sequence
hsa_circ_0123248 (circDLG1)-1	5'-AACATACGTTATTCACCGATATAAT-3'
hsa_circ_0123248 (circDLG1)-2	5'-CGTTATTCACCGATATAATACGATC-3'
hsa_circ_0123248 (circDLG1)-3	5'-TACGTTATTCACCGATATAATACGA-3'
mmu_circ_0000679 (circDlg1)-1	5'-AAACACACACTGTTCACCGATATGA-3'
mmu_circ_0000679 (circDlg1)-2	5'-CTAAACACACACTGTTCACCGATAT-3'
mmu_circ_0000679 (circDlg1)-3	5'-AACACACACTGTTCACCGATATG-3'

RNA pull down probes	Sequence
NC probe	5'-BiotinAAAAAAAAAAAAAAAAAAAAAAAA-3'
mmu_circ_0000679 (circDlg1)-1	5'-BiotinGGTCCGCCAGCAAGGATGAAGGAGATAAAA-3'
mmu_circ_0000679 (circDlg1)-2	5'-BiotinGCAGGAGGACGGCTGACATGGTTG-3'
mmu_circ_0000679 (circDlg1)-3	5'-BiotinTTCCCTAGTGATCTCGTCATCTCCG-3'

siRNAs	Sequence
si-mmu_circ_0000679 (si-circDlg1)	5'-AUCGGUGAACAGUGUGUGUTT-3' (sense);
	5'-ACACACACUGUUCACCGAUTT-3' (anti-sense)
si-hsa_circ_0123248 (si-circDLG1)	5'-AUCGGUGAAUAACGUAUGUTT-3' (sense);
	5'-ACAUACGUUAUUCACCGAUTT-3' (anti-sense)
si-PDE4B	5'-CAAUGUGGCUGGGUACUCATT-3' (sense);
	5'-UGAGUACCCAGCCACAUUGTT-3' (anti-sense)

qPCR primers	Sequence
Actb	5'-GTCATCACTATTGGCAACGAGC-3' (forward);
	5'-TTGGCATAGAGGTCTTTACGGAT-3' (reverse)
Aif1	5'-CGAATGCTGGAGAAACTTGG-3' (forward);
	5'-GCCTCTTGTGTTCTTTGTTTTTC-3' (reverse)
ApoE	5'-TCGGGCAGTACCGCAACG-3' (forward);
	5'-GCTCACGGATGGCACTCACA-3' (reverse)
Arg1	5'-GGATTGGCAAGGTGATGG-3' (forward);
	5'-AAGGAGCCCTGTCTTGTAAAT-3' (reverse)
Axl	5'-GAGCCAACCGTGGAAAGAG-3' (forward);
	5'-CCACCTTATGCCGATCTACC-3' (reverse)
CD206	5'-GGCAGTGGGCTGGAGGAA-3' (forward);
	5'-TAGGCACATCGCTTGCTGAG-3' (reverse)
CD86	5'-GCTTTGACAGGAACAACTGGACTC-3' (forward);
	5'-TCGGGTGACCTTGCTTAGACG-3' (reverse)
Clec7a	5'-CTCAGCCTTGCCTTCCTAAT-3' (forward);
	5'-ATACGGTGAGACGATGTTTGG-3' (reverse)

Cst7	5'-TATGCTGGAGGTGAAAATCGG-3' (forward);
	5'-TGTGGAGCCAGGGGATGAC-3' (reverse)
Cx3cr1	5'-TTGCCTCAACCCCTTTATCTA-3' (forward);
	5'-GCTGTCCTGCCTGCTCCT-3' (reverse)
Dlg1	5'-ATCTATTGTGCGATTGTATGTGA-3' (forward);
	5'-ATGCTGTTATCACCAGGAATG-3' (reverse)
GAPDH	5'-CATCACTGCCACCCAGAAGA-3' (forward);
	5'-GGACACATTGGGGGTAGGA-3' (reverse)
GFAP	5'-GGAGGGCGAAGAAAACCG-3' (forward);
	5'-TCTCCACAGTCTTTACCACGATG-3' (reverse)
hsa_circ_0123248 (circDLG1)	5'-GGAGGAGAAGATGGAGAAGGA-3' (forward);
	5'-CCACTTTCAAATAAACAAAATCAG-3' (reverse)
IL-1β	5'-AAATCTCGCAGCAGCACAT-3' (forward);
	5'-ATGAGTCACAGAGGATGGGC-3' (reverse)
IL-6	5'-CTTGGGACTGATGCTGGTGA-3' (forward);
	5'-ACTCTTTTCTCATTTCCACGATTT-3' (reverse)
iNOS	5'-GTTTACCATGAGGCTGAAATCC-3' (forward);
	5'-CCTCTTGTCTTTGACCCAGTAG-3' (reverse)
Lpl	5'-ACTGAGGATGGCAAGCAACAC-3' (forward);
	5'-ATGAGCAGTTCTCCGATGTCC-3' (reverse)
mmu_circ_0000108	5'-ACTTCTTCAATGATTTTCACCTC-3' (forward);
	5'-TGGACATTTCTCTTGTTAGCAG-3' (reverse)
mmu_circ_0000203	5'-GCTGAGGGGGACAGAATC-3' (forward);
	5'-TTAGGAGGTCGCAAGGTGA-3' (reverse)
mmu_circ_0000204 (circAnks1b)	5'-AAGTCCAACCACCACTACTGTCA-3' (forward);
	5'-GCTTCATTAGGAGGTCGCAA-3' (reverse)
mmu_circ_0000378	5'-AGGCAAATCAAACGGCAAC-3' (forward);
	5'-GGCTTCCTTGAGGGCACA-3' (reverse)
mmu_circ_0000387	5'-CTCTTAGGACGGCTTGGACG-3' (forward);
	5'-AGGAGCAGAGCAACAGGGAG-3' (reverse)
mmu_circ_0000609	5'-GAGAGTATGACTATGACGATGGGTA-3' (forward);
	5'-TGCCAAGGATGGACATTTTT-3' (reverse)
mmu_circ_0000679 (circDlg1)	5'-TCTCCTTCATCCTTGCTGG-3' (forward);
	5'-CACTTTCAAATAAACAAAATCAGA-3' (reverse)
mmu_circ_0001115	5'-TTGCCTGTGATGAGAACCCG-3' (forward);
	5'-ACTCCTCTTTCAATGTGTTGCCTT-3' (reverse)
mmu_circ_0001751 (circCarm1)	5'-CTACCTATCCCAGCAGCAGA-3' (forward);
	5'-CAGCCCAGGGTGATGAT-3' (reverse)
P2ry12	5'-AACCATTGACCGCTACCTGA-3' (forward);
	5'-CATTTTGTTACGTCCTTATCTTTTG-3' (reverse)
h-PDE4B	5'-TAGTCAGCCTCCTGTCTCCAGA' (forward);
	5'-GAAGCCATCTCACTGACAGACC-3' (reverse)
PDE4B1	5'-CAGAGTGAAAGGGCAAGGACC-3' (forward);
	5'-AGTCCCGACGAAGAGCCG-3' (reverse)

PDE4B2 (PDE4B)	5'-ATGGAGACGCTGGAGGAACTA-3' (forward);
	5'-GTGTGTCAGCTCCCGGTTC-3' (reverse)
PDE4B3	5'-CGTCGCTTCACGGTGGC-3' (forward);
	5'-TCCTGGACATCGCTTTTGGT-3' (reverse)
PDE4B5	5'-GCCTGAGGCAAACTATTTATTATC-3' (forward);
	5'-CCACATCGTTCTGCTTGTCTAA-3' (reverse)
Tmem119	5'-CGTGCCACCCACCAACCT-3' (forward);
	5'-CATACTTCTTTTCAGGGAACGAGG-3' (reverse)
TNF-α	5'-GAGTGACAAGCCTGTAGCCC-3' (forward);
	5'-TTGTCCCTTGAAGAGAACCTG-3' (reverse)
Trem2	5'-TAGCCTACCACCTTCCTCCTCTT-3' (forward);
	5'-GCTTCTGCCTGCCCCTG-3' (reverse)
Tyrobp	5'-TCTTCCGTGAGCCCTGGTGTA-3' (forward);
	5'-TCCCTTCCGCTGTCCCTTG-3' (reverse)
